# Isolation, structural characterization of oligosaccharide AMO-1 from *Avicennia marina* fruit and its anti-osteoporotic effects via PI3K/AKT/HIF‑1α signaling pathway activation

**DOI:** 10.1007/s13659-026-00646-8

**Published:** 2026-10-22

**Authors:** Lishuang Zhou, Zhenhui Feng, Yijing Zhang, Lei Tan, Pan Zhou, Xia Wu, Xing Chen, Qinyuan Zhang, Qiwen Cai, Liutong Yu, Kefeng Wu, Luming Deng

**Affiliations:** 1 The Marine Biomedical Research Institute of Guangdong Zhanjiang, School of Ocean and Tropical Medicine, Guangdong Medical University, Zhanjiang, 524023 People’s Republic of China; 2 Guangdong Engineering Technology Research Center for the Development and Utilization of Mangrove Wetland Medicinal Resources, Guangdong Medical University, Zhanjiang, 524023 People’s Republic of China; 3 College of Tropical Southern Medicinal Herbs and Marine Organism Utilization, Guangdong Medical University, Zhanjiang, 524023 People’s Republic of China; 4 Department of Environmental Science and Engineering, Fudan University, Shanghai, 200438 People’s Republic of China

**Keywords:** *Avicennia marina*, Mangrove plant, Oligosaccharide, Structural characterization, Anti-osteoporosis

## Abstract

*Avicennia marina* (Forssk.) Vierh. is an important mangrove plant with high nutritional and medicinal values, featuring saccharides as its primary bioactive compounds. In this study, we successfully isolated a neutral tetrasaccharide (AMO-1) from the fruits of *A. marina*. Through monosaccharide composition, methylation, UPLC-ESI–MS/MS, and NMR analyses, we revealed that the structure of AMO-1 was α-D-Gal*p*-(1 → 6)-α-D-Gal*p*-(1 → 6)-α-D-Glc*p*-(1 → 2)-β-D-Fru*f*. Subsequently, we investigated the anti-osteoporosis (anti-OP) efficacy and underlying mechanisms of AMO-1. Our findings demonstrated that AMO-1 promoted the differentiation and mineralization of MC3T3-E1 cells. Furthermore, AMO-1 increased trabecular bone density and restored its reticular structure in dexamethasone-induced OP mice. Additionally, AMO-1 upregulated the expression levels of osteogenic factors and elevated serum levels of osteocalcin and PINP. Mechanistically, AMO-1 promoted osteogenesis through activation of the PI3K/AKT/HIF-1α signaling pathway, thus exerting anti-OP effects. Overall, these findings indicate that AMO-1 is a promising functional food ingredient with therapeutic potential for the prevention and management of OP.

**Graphical Abstract:**

**Figure:**
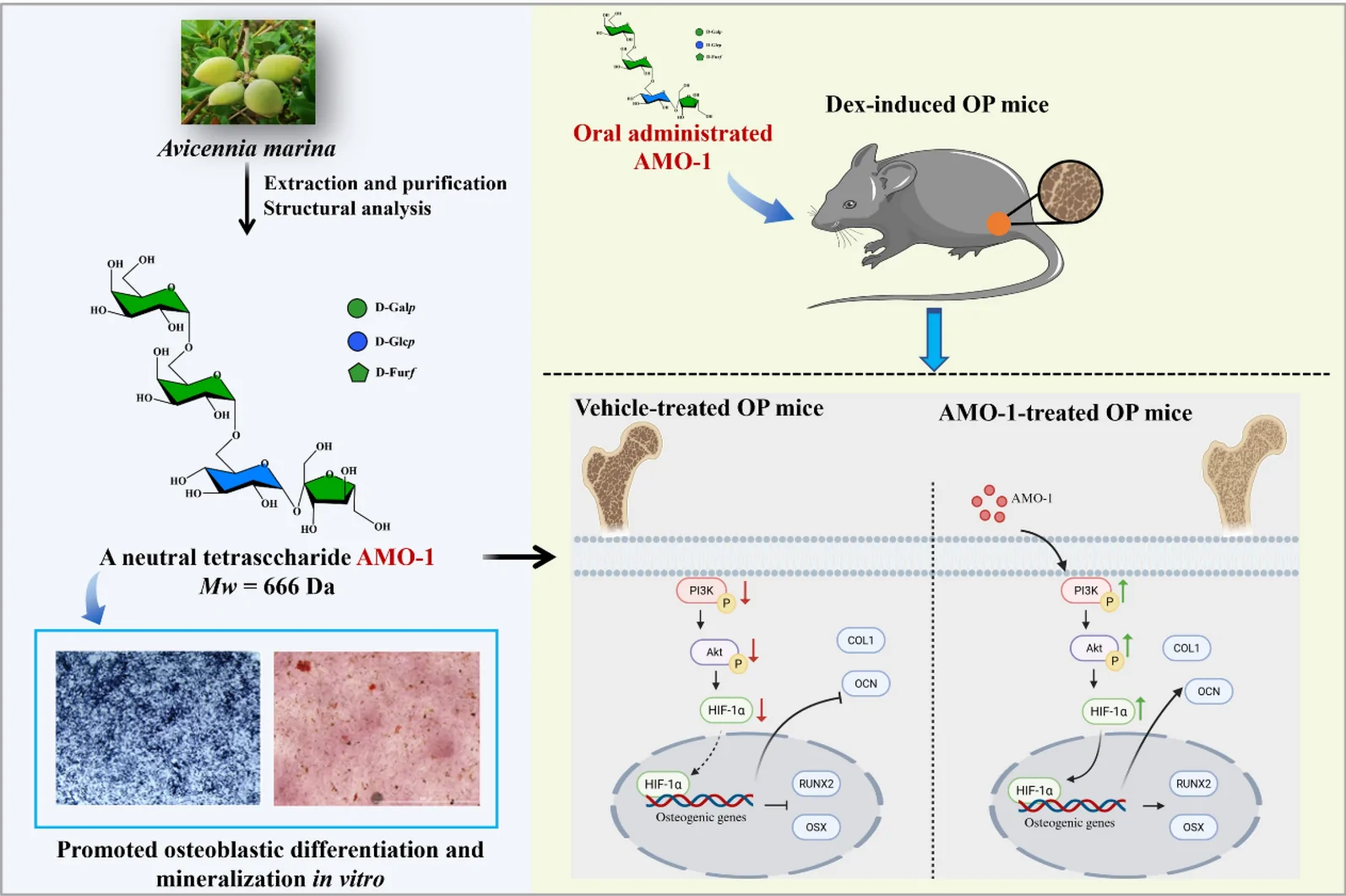

**Supplementary Information:**

The online version contains supplementary material available at https://doi.org/10.1007/s13659-026-00646-8.

## Introduction

Osteoporosis (OP) is recognized as one of the most prevalent metabolic disorders that impact the skeletal system [1]. As the world’s population continues to age rapidly, the incidence of OP has escalated, making it a considerable public health challenge. This condition not only detracts from the quality of life for those affected but also imposes a substantial economic strain on healthcare systems and society as a whole [2]. It is crucial to emphasize that the maintenance of bone homeostasis relies on a consistent equilibrium between bone formation driven by osteoblasts and bone resorption carried out by osteoclasts. An imbalance between osteoblasts and osteoclasts is regarded as one of the primary causes of OP [3]. Currently, the medications available for preventing and treating OP are mainly divided into anti-resorptive agents and anabolic agents. Unfortunately, the extended usage of these treatments has been associated with several adverse effects [4]. Consequently, there is an urgent need to identify pharmacological alternatives that are safer and more effective for preventing and managing OP. Over the long term, natural products generally exhibit fewer toxic side effects, making them promising candidates for alternative or supplementary treatments.

*Avicennia marina* (Forssk.) Vierh., which is part of the *Acanthaceae* family, is a mangrove species renowned for its therapeutic and nutritional uses [5]. This species plays an important ecological role and is employed in traditional medicine to treat a variety of health issues, including ulcers, smallpox, and rheumatism, in numerous Asian and African nations, including China, Pakistan, and Egypt [6]. Among the major bioactive compounds in *A. marina*, saccharides exhibit a diverse range of biological functions, including anticomplementary, antimicrobial, probiotic, and immunomodulatory activities. Therefore, these substances have attracted considerable interest from researchers in recent years. A prior study revealed that an acidic polysaccharide known as HAM-3-IIb-II, isolated from *A. marina*, significantly affected the lipopolysaccharide-induced proliferation of B lymphocytes [7]. Additionally, polysaccharides AMFPs obtained from the fruit of *A. marina* significantly reduced harmful bacteria, increased certain beneficial bacteria, and significantly elevated various short-chain fatty acid concentrations. These results indicate that AMFPs may serve as effective prebiotics to enhance gut health [8]. However, the anti-OP properties of saccharides isolated from *A. marina*, along with the mechanisms behind these effects, have yet to be reported.

Numerous investigations have shown that the suppression of bone formation is a significant contributor to the pathogenesis of OP, with osteoblasts being integral to this mechanism [9,10]. Prior research has indicated that glucocorticoids can impair the process of bone formation by affecting osteoblasts' proliferation, differentiation, and mineralization [11]. The PI3K/AKT signaling cascade regulates several biological functions within cells, including proliferation, survival, apoptosis, and metabolism. Research has demonstrated that this signaling pathway is vital to the progression of OP. In China, numerous traditional herbal medications have been employed for centuries to prevent and treat OP [12]. The primary bioactive constituents of these traditional therapies are natural saccharides, with several exhibiting potential anti-OP effects through the modulation of the PI3K/AKT signaling pathway. For example, polysaccharide EBP, derived from *Epimedium brevicornum*, prevented osteoblast apoptosis by enhancing the PI3K/AKT/mTOR signaling pathway, exerting an osteoprotective effect against glucocorticoid-induced OP in vitro [13]. Additionally, the polysaccharide DAP, isolated from the roots of *Dipsacus asper*, reduced bone loss resulting from ovariectomy in rats by stimulating the PI3K/AKT/eNOS signaling pathway [14]. The protein known as hypoxia-inducible factor-1α (HIF-1α) functions as a downstream component within the PI3K/AKT signaling pathway and is vital for sustaining bone homeostasis, as well as being involved in several bone-related conditions, such as OP [15]. Previous research has indicated that increasing HIF-1α expression enhances the osteogenic capability of MC3T3-E1 cells. Additionally, HIF-1α serves as a transcription factor that modulates the production of vascular endothelial growth factor (VEGF), leading to an enhancement in trabecular bone volume and a boost in bone vascular density [16]. Importantly, HIF-1α has also been found to facilitate fracture repair, increase bone mineral density, and fortify bone strength by elevating the expression of proteins associated with osteogenic differentiation, such as RUNX2, OSX, and COL1 [17]. Consequently, osteogenic therapeutics that stimulate the PI3K/AKT/HIF-1α signaling cascade could represent an effective approach for treating OP.

In the present study, an oligosaccharide known as AMO-1 was isolated from the fruits of *A. marina* through DEAE-cellulose 52 and Sephacryl S-200 column chromatography methods. The structural characteristics of AMO-1 were determined using techniques such as monosaccharide composition, methylation, UPLC-ESI–MS/MS, and NMR analyses. Additionally, both in vitro and in vivo assessments of AMO-1's anti-OP effects were conducted using MC3T3-E1 cells and a mouse model of OP induced by dexamethasone (DEX). Finally, the potential mechanism by which AMO-1 exerted its anti-OP activity was elucidated through proteomic analysis combined with Western blot experiments. This investigation may provide valuable insights into the potential application of AMO-1 as a functional ingredient for the prevention and management of OP.

## Results

### Isolation, purification, and general properties of AMO-1

**Fig. 1:**
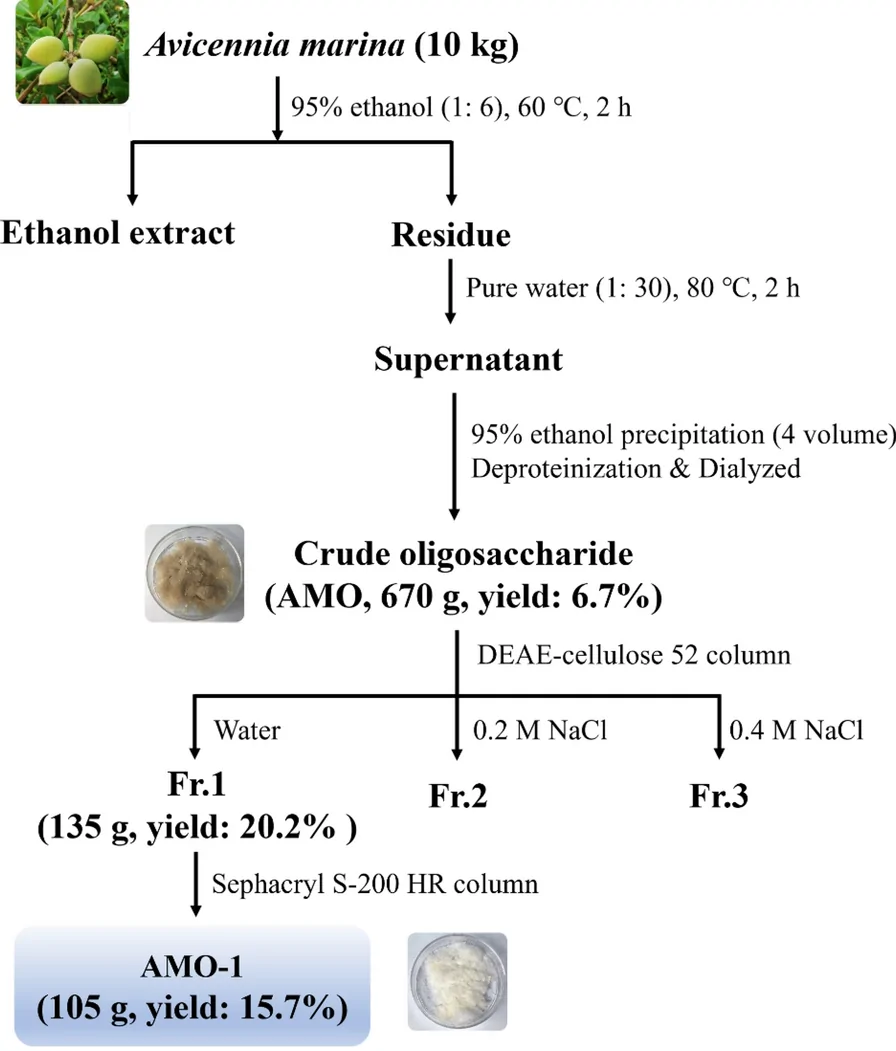
Isolation procedure of oligosaccharide AMO-1 from *A. marina*

**Fig. 2:**
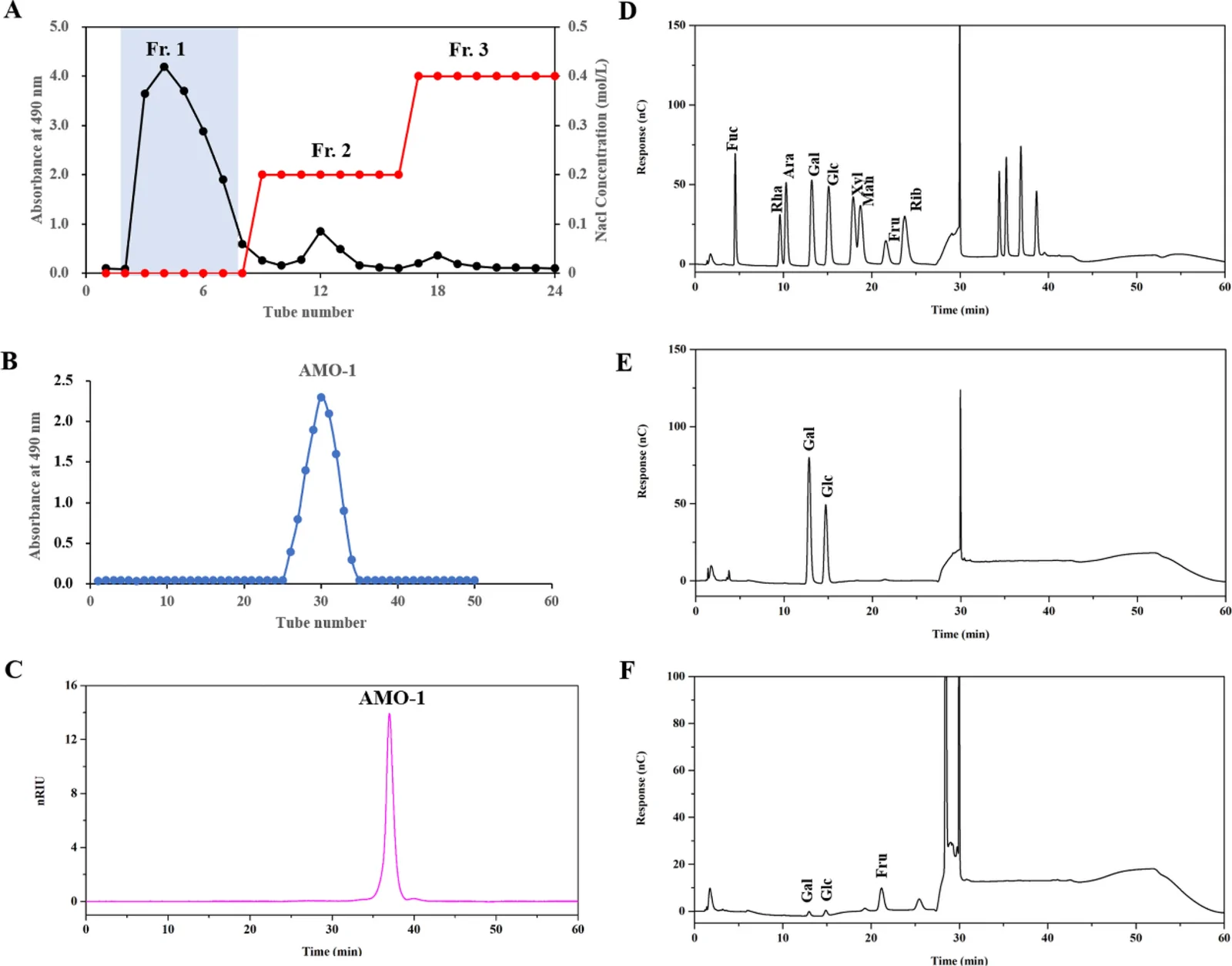
**A** Elution curve AMO on DEAE-cellulose 52 column. **B** Elution curve AMO-1 on Sephacryl S-200 column. **C** HPGPC results for AMO-1. Monosaccharide compositions for the standards (**D**) and AMO-1 (E&F)

**Fig. 3:**
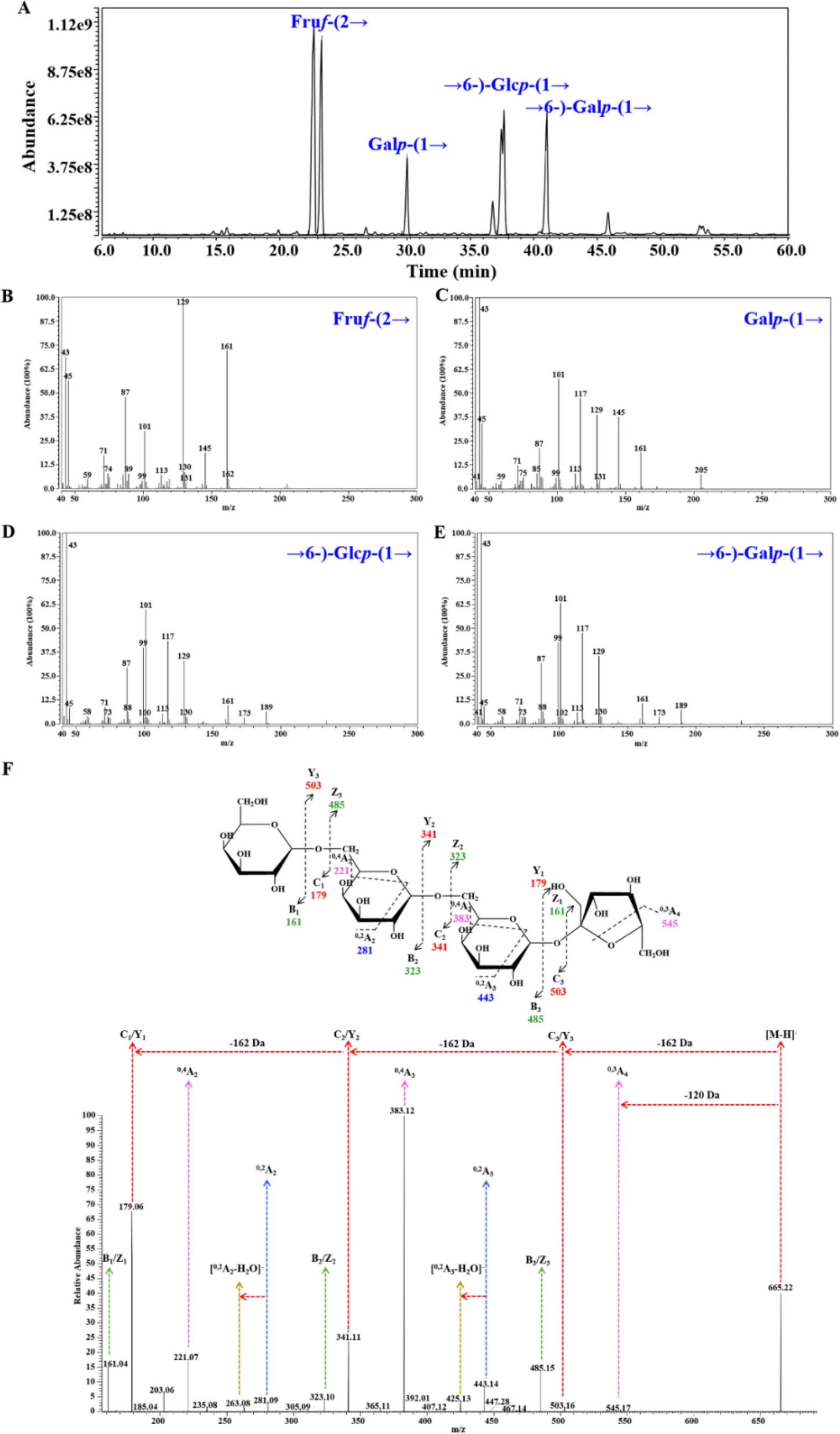
The total ion chromatograms (**A**) alongside the related mass spectra (**B**–**E**) for PMAAs in AMO-1. The UPLC-ESI–MS/MS spectra and the fragmentation pathways of AMO-1 (**F**)

The water-soluble saccharide fraction AMO was isolated from *A. marina* fruits via hot water extraction, ethanol precipitation and deproteinization, with a yield of 6.70% relative to raw materials (Fig. 1). For further purification, DEAE-cellulose 52 column chromatography combined with Sephacryl S-200 gel filtration was applied, yielding the purified oligosaccharide AMO-1 with a yield of 15.7% relative to crude AMO (Fig. 2A, B). The HPGPC profile illustrated in Fig. 2C for AMO-1 displayed a single symmetrical peak, suggesting that it is a homogeneous oligosaccharide. A significant carbohydrate composition of 98.80% further confirmed a high degree of purity. Negative ion mode analysis performed using UPLC-ESI–MS/MS revealed a peak at m/z 665, corresponding to the [M-H]^−^ ion, which indicates that the molecular weight of AMO-1 is 666 Da (Fig. 3F). Additionally, the UV spectrum did not show any distinctive absorption at 260 nm or 280 nm, which indicates the absence of both nucleic acids and proteins (Fig. S1).

### Monosaccharide composition analysis

**Fig. 4:**
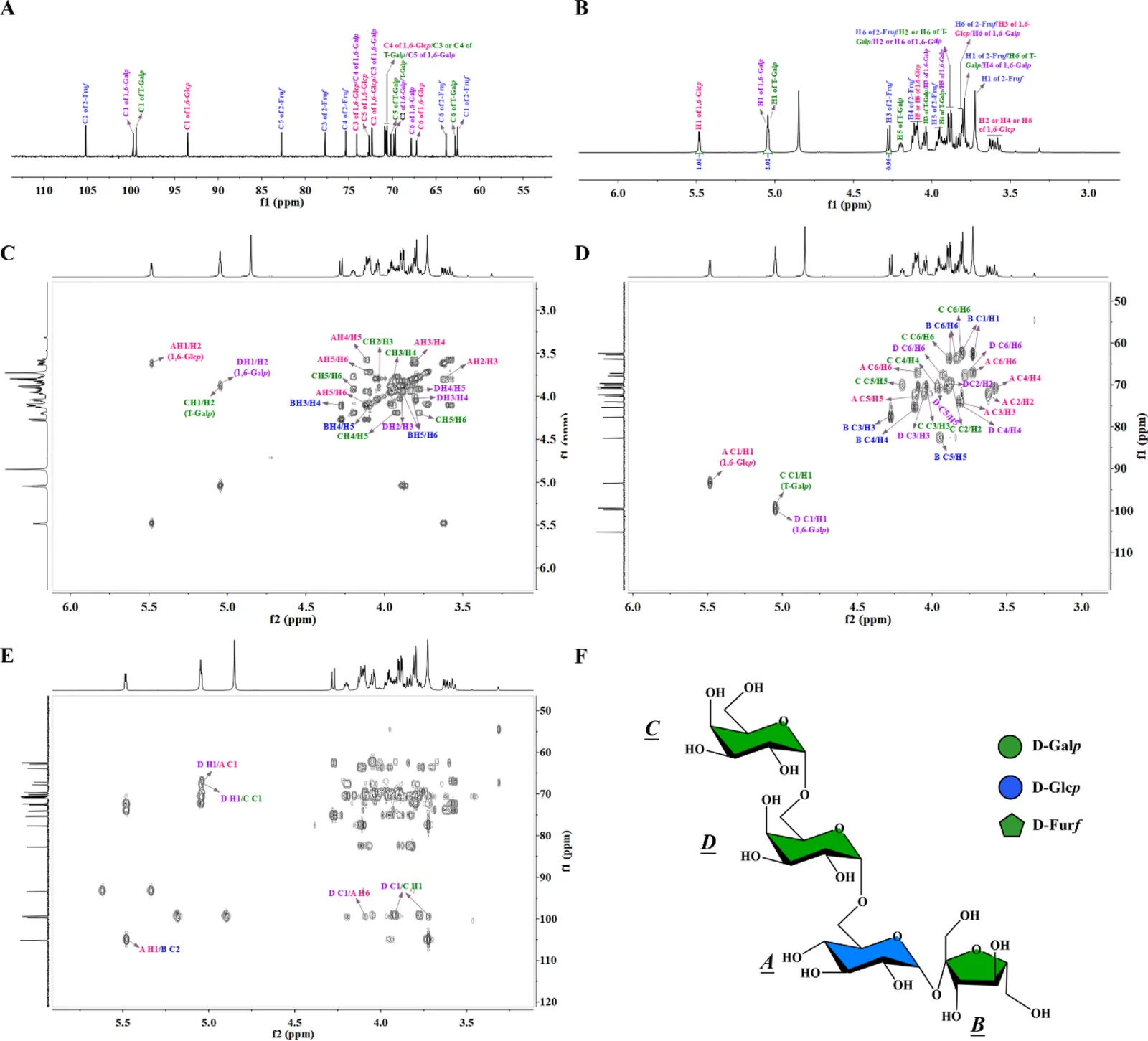
**A**–**E** The ^13^C-NMR, ^1^H-NMR, HSQC, ^1^H-^1^H COSY, and HMBC spectra of AMO-1; **F** The precise structure of AMO-1

The quantitative analysis of monosaccharide composition in AMO-1 was conducted using ion chromatography. The chromatographic profiles displayed in Fig. 2D–F reveal that AMO-1 is composed of three monosaccharide residues: galactose (Gal), Glc, and Fru, which are present in a molar ratio of 0.25: 0.16: 0.15 (see detailed calculations in the Supplementary Information). Coupled with the determination of molecular weight (666 Da) via UPLC-ESI–MS/MS analysis, this stoichiometric ratio provided strong evidence for the structural identification of AMO-1 as a tetrasaccharide consisting of two Gal residues, one Glc residue, and one Fru residue (Fig. 3C). To further confirm the stoichiometry of the residues, we conducted ^1^H-NMR spectroscopy. The ^1^H-NMR spectrum exhibited clear proton signals at δ 5.04–5.05 ppm (Gal H-1), δ 5.48 ppm (Glc H-1), and δ 4.27 ppm (Fru H-3), with signal integration ratios consistent with the proposed molar composition (Fig. 4B) [18].

### FT-IR spectrum analysis

The examination of the FT-IR spectrum depicted in Fig. S2 facilitated the recognition of the distinct functional groups within AMO-1. There was a prominent and pronounced stretching peak at 3395 cm^−1^, attributed to the O–H stretching vibration within the sugar residues present in AMO-1. Moreover, the peaks at 2919 cm^−1^ and 1416 cm^−1^ were associated with the bending and stretching vibrations of C–H bonds, respectively. Another peak identified at 1645 cm^−1^ was connected to the C = O stretching vibration. The peaks at 1244 cm^−1^ and 1045 cm^−1^ revealed the presence of C–O–C and C–O–H linkages. Finally, the peaks recorded at 771 cm^−1^ and 536 cm^−1^ suggested that α- and β-glycosidic linkages were simultaneously present in the oligosaccharide AMO-1 [19].

### Methylation analysis

Based on the existing literatures [18,20] and the GC–MS datasets pertaining to PMAAs, we discovered a total of five distinct peaks within the total ion chromatograms (TIC) for AMO-1 (Fig. 3A). These peaks correspond to four different PMAAs, which include 1,3,4,6-tetra-*O*-methyl-(2-deuterio)-2,5-di-*O*-acetyl hexitol (mannitol, glucitol), 1,5-Di-*O*-acetyl-1-deuterio-2,3,4,6-tetra-*O*-methyl-D-galactitol, 1,5,6-Tri-*O*-acetyl-1-deuterio-2,3,4-tri-*O*-methyl-D-glucitol, and 1,5,6-Tri-*O*-acetyl-1-deuterio-2,3,4-tri-*O*-methyl-D-galactitol, respectively (Fig. 3B–E). The glycosidic linkages related to these were determined to be Fru*f*-(2 → , Gal*p*-(1 → , → 6-Glc*p*-(1 → , and → 6)-Gal*p*-(1 → , with approximate molar ratios of 0.23: 0.08: 0.11: 0.09 (Table 1).

**Table 1:** Methylation analysis of AMO-1

PMAAs	Type of linkage	Molar ratios	Mass fragments (m/z)
1,3,4,6-tetra-*O*-methyl-(2-deuterio)-2,5-di-*O*-acetyl hexitol (mannitol, glucitol)	Fru*f*-(2 →	0.23	87,101,129,143,161
1,5-Di-*O*-acetyl-1-deuterio-2,3,4,6-tetra-*O*-methyl-D-galactitol	Gal*p*-(1 →	0.08	43,71,87,101,117,129,143,161,205
1,5,6-Tri-*O*-acetyl-1-deuterio-2,3,4-tri-*O*-methyl-D-glucitol	→ 6-Glc*p*-(1 →	0.11	43,87,99,101,117,129,161,189,233
1,5,6-Tri-*O*-acetyl-1-deuterio-2,3,4-tri-*O*-methyl-D-galactitol	→ 6)-Gal*p*-(1 →	0.09	43,87,99,101,117,129,161,189,233

### UPLC-ESI–MS/MS for identification of AMO-1 structure

To attain a more comprehensive understanding of the structural characterization of AMO-1, UPLC-ESI–MS/MS analysis was conducted [20]. As detailed in Fig. 3F, the precursor ion [M-H]^−^ at *m/z* 665 was fragmented, yielding C_3_/Y_3_-C_1_/Y_1_ type ions (*m/z* 503, 341, 179), B_3_/Z_3_-B_1_/Z_1_ type ions (*m/z* 485, 323, 161), [^0,2^A_3_-H]^−^-[^0,2^A_2_-H]^−^ ions (*m*/*z* 443, 281), [^0,4^A_3_-H]^−^-[^0,4^A_2_-H]^−^ ions (*m*/*z* 383, 221), [^0,2^A_3_-H-H_2_O]^−^-[^0,2^A_2_-H-H_2_O]^−^ ions (*m*/*z* 425, 263), as well as [^0,3^A_4_-H]^−^ ions (*m*/*z* 545). The structure of AMO-1 was distinctly clarified as Gal ^1^ → ^6^ Hex ^1^ → ^6^ Hex ^1^ → ^2^ Fru, based on characteristic neutral losses and cross-ring cleavage behavior, together with the findings from methylation analysis.

### NMR analysis

NMR spectroscopy was employed as a powerful analytical technique to examine the structure of glycosidic bonds and to explore the interrelationships among glycosidic residues within the oligosaccharide. The resulting data from the ^1^H and ^13^C NMR spectra, along with 2D NMR spectra of AMO-1, are displayed in Fig. 4.

In the ^13^C NMR spectrum depicted in Fig. 4A, three prominent anomeric peaks at δ 93.60, δ 99.40, and δ 99.72 correspond to the C-1 of → 6)-α-D-Glc*p*-(1 → (**A**), α-D-Gal*p*-(1 → (**C**), and → 6)-α-D-Gal*p*-(1 → (**D**), respectively. Additionally, the downfield signal at δ 105.17 is linked to the C-2 of → 2)-β-D-Fru*f* (**B**) [20]. The corresponding chemical shifts of the anomeric protons were further elucidated by the HSQC spectrum (Fig. 4C) in conjunction with the ^1^H-^1^H COSY spectrum (Fig. 4D), yielding values of 5.48 ppm (**A**), 5.04 ppm (**C**), and 5.05 ppm (**D**). In the DEPT-135 NMR spectrum (Fig. S3), the negative peak observed at δ 62.48 corresponds to the C-1 position of → 2)-β-D-Fru*f*. The inverted peaks at δ 62.81, δ 63.83, δ 67.19, and δ 67.83 are attributed to the C-6 of α-D-Gal*p*-(1 → , → 2)-β-D-Fru*f*, → 6)-α-D-Glc*p*-(1 → , and → 6)-α-D-Gal*p*-(1 → , respectively. Furthermore, the pronounced signals at δ 77.78, δ 75.42, and δ 82.88 correspond to the C-3, C-4, and C-5 of → 2)-β-D-Fru*f*, respectively. Resonances found at δ 69.83, δ 70.67, δ 70.58, and δ 70.14 relate to the C-2, C-3, C-4, and C-5 of → 6)-α-D-Gal*p*-(1 → , respectively. Additional signals can be found in Table 2.

**Table 2:** ^1^H and ^13^C NMR chemical shifts of AMO-1

Glycosyl residues	H1/C1	H2/C2	H3/C3	H4/C4	H5/C5	H6/C6
→ 6)-α-D-Glc*p*-(1 →	5.48	3.62	3.80	3.59	4.12	3.63/4.09
Residue A	93.60	72.23	74.07	70.81	72.68	67.19
→ 2)-β-D-Fru*f*	3.72/3.79	−	4.27	4.10	3.95	3.83/3.88
Residue B	62.48	105.17	77.78	75.42	82.88	63.83
α-D-Gal*p*-(1 →	5.04	3.87	4.04	3.94	4.19	3.78/3.91
Residue C	99.40	69.83	70.67	70.58	70.14	62.81
→ 6)-α-D-Gal*p*-(1 →	5.05	3.89	4.05	3.81	3.94	3.78/3.91
Residue D	99.72	69.67	72.32	74.13	70.93	67.83

The glycosyl residue sequences of AMO-1 were identified using the HMBC spectrum (Fig. 4E). The presence of cross peaks observed at δ 99.40/3.78/3.91 (**C** C1/ **D** H6) and δ 5.04/67.83 (**C** H1/ **D** C6) suggests that residue **C** is connected to residue **D** via a (1 → 6) linkage. Meanwhile, the cross peaks at δ 99.72/3.63 (**D** C1/ **A** H6) and δ 5.05/67.19 (**D** H1/ **A** C6) indicate that residue **D** is connected to residue **A** through a (1 → 6) bond. Furthermore, the cross peaks at δ 5.48/105.17 (**A** H1/ **B** C2) illustrate the connection between residue **A** and residue **B** also through a (1 → 6) linkage.

Considering the analysis of monosaccharide composition, methylation, UPLC-ESI–MS/MS, and NMR, the exact structure of AMO-1 has been delineated. This structure has been identified as α-D-Gal*p*-(1 → 6)-α-D-Gal*p*-(1 → 6)-α-D-Glc*p*-(1 → 2)-β-D-Fru*f*. A structural representation of AMO-1 is illustrated in Fig. 4F.

### Morphological analysis

SEM is a widely utilized technique in the examination of the architecture of macromolecules, with polysaccharides being one of the primary subjects of study. Fig. S4 displays the SEM images of AMO-1 at magnifications of 500 × and 2000 × . The appearance of AMO-1 resembles sheets of varying dimensions, characterized by a frosted surface, aggregation folding, and a loosely stacked arrangement.

### Cell viability assay

To evaluate the cytotoxic effects of AMO-1, varying doses of AMO-1 were administered to MC3T3-E1 cells in order to examine its influence on cell viability. The CCK-8 assays revealed no cytotoxic effects at any concentration up to 100 μg/mL (Fig. S5). As a result, the doses of 12.5, 25, and 50 μg/mL of AMO-1 were chosen for additional analysis concerning their influence on the differentiation and mineralization of MC3T3-E1 cells.

### AMO-1 enhanced osteogenic differentiation and mineralization in MC3T3-E1 cells

**Fig. 5:**
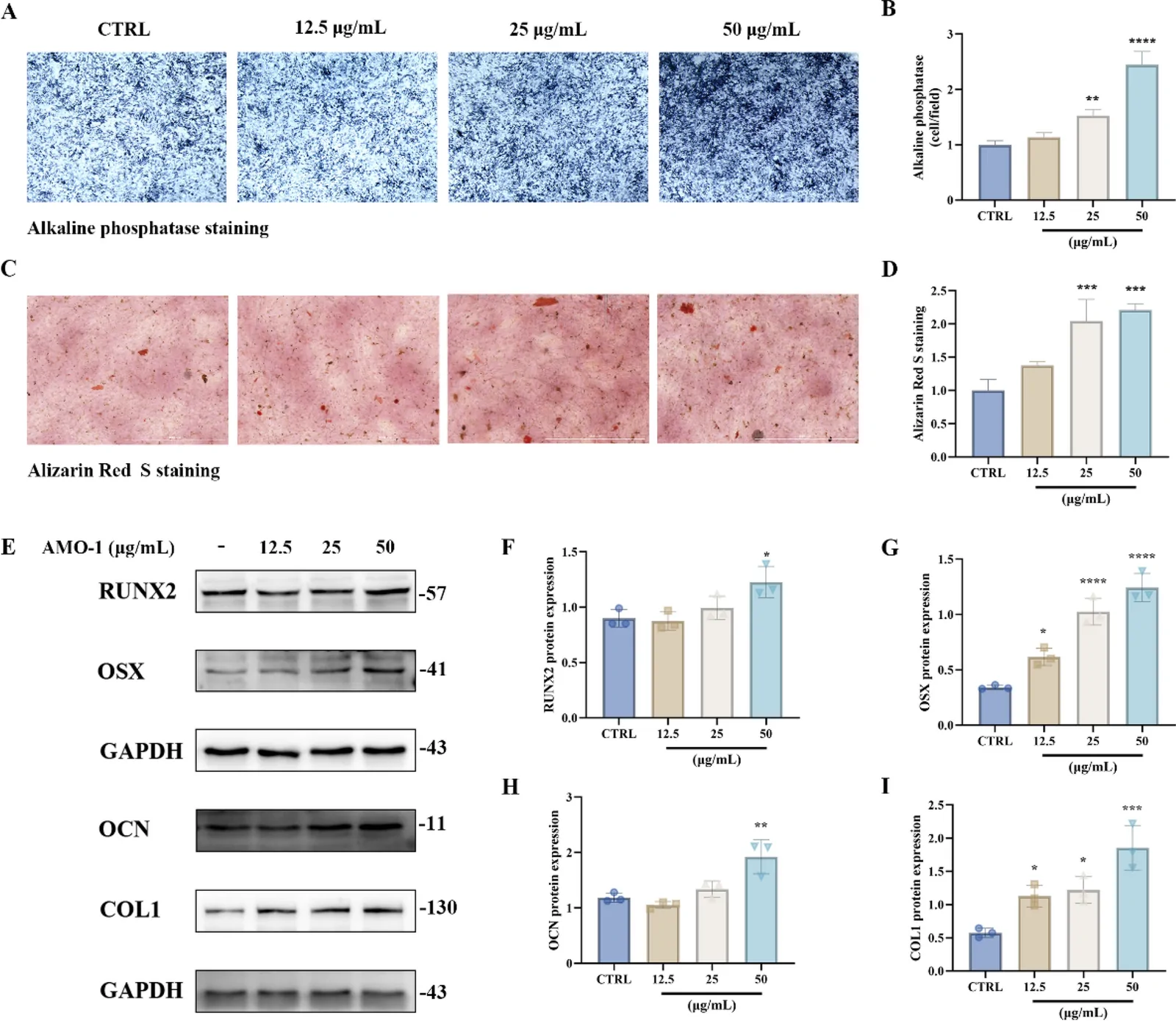
**A** Staining images for ALP (10 ×). **B** Measurement of areas stained for ALP. **C** Staining images for ARS at 10 × magnification. **D** Measurement of areas stained for ARS. **E** Analysis via Western blotting of the levels of RUNX2, OSX, OCN, and COL1 proteins in MC3T3-E1 cells. **F** Measurement of RUNX2. **G** Measurement of OSX. **H** Measurement of OCN. **I** Measurement of COL1. *p < 0.05, **p < 0.01, ***p < 0.001, ^****^p < 0.0001 vs. CTRL, n = 3

Results from ALP staining experiments revealed that treatment with AMO-1 resulted in a notable increase in the number of cells positive for ALP staining compared to the CTRL group (Fig. 5A). Furthermore, after a 7-day treatment period, AMO-1 at doses of 25 and 50 μg/mL significantly enhanced ALP activity relative to the CTRL group (Fig. 5B). These results indicate that AMO-1 considerably enhances the osteogenic differentiation of MC3T3-E1 cells. Additionally, the influence of AMO-1 on the osteogenic mineralization process in MC3T3-E1 cells is illustrated in Fig. 5C, D. Following 14 days of treatment, the groups treated with AMO-1 showed a dose-dependent augmentation in the areas exhibiting red coloration when compared to the CTRL group, indicating that AMO-1 enhances calcium deposition in MC3T3-E1 cells. These findings support the conclusion that AMO-1 facilitates osteogenic mineralization in vitro.

### AMO-1 enhanced the levels of osteogenesis-related proteins in MC3T3-E1 cells

Proteins involved in osteogenesis, such as RUNX2, OSX, OCN, and COL1, are crucial for the differentiation of osteoblasts and the entire process of bone formation. To evaluate the impact of AMO-1 on the osteogenic capabilities of MC3T3-E1 cells, we performed a protein expression analysis utilizing the Western blot technique, as demonstrated in Fig. 5E. The results, presented in Fig. 5F and H, revealed that administering AMO-1 at a dosage of 50 μg/mL significantly elevated the protein levels of RUNX2 and OCN compared to the CTRL group. Additionally, the levels of COL1 and OSX proteins demonstrated a dose-dependent augmentation in the AMO-1 treated groups relative to the CTRL group (Fig. 5G and I). In conclusion, the elevation of these proteins associated with osteogenesis highlighted the enhanced osteogenic differentiation of MC3T3-E1 cells, aligning with the results obtained from the ALP and ARS staining assessments.

### AMO-1 attenuated microarchitecture degradation of femoral tissue in OP mice

To further validate the in vivo anti-OP effects of AMO-1, we evaluated its influence on a mouse model that was induced with OP using DEX. As detailed in Table S1, continuous treatment with DEX led to a notable decline in body weight, whereas the liver index demonstrated an increase in OP mice when compared to the CTRL group. Conversely, administration of AMO-1 brought about significant improvements in the liver index, even though it did not have a considerable impact on the body weight of OP mice. Notably, after 63 days of exclusive AMO-1 treatment, healthy mice did not exhibit any notable changes in body weight or organ indices (Table S1). H&E staining analyses revealed that there were no significant pathological changes in liver tissues across the various groups (Fig. S6A). In contrast, the spleens of vehicle-treated OP mice lacked a clear difference between red and white pulp, accompanied by slight infiltration of inflammatory cells (Fig. S6B). Furthermore, the kidneys exhibited signs of glomerular atrophy and tubular vacuolar degeneration (Fig. S6C), both of which were effectively reversed following treatment with AMO-1 (Fig. S6B, C). Importantly, treatment with AMO-1 alone did not result in any adverse effects on the structural integrity of organs in healthy mice, highlighting the compound's biological safety, as evidenced in Fig. S6.

**Fig. 6:**
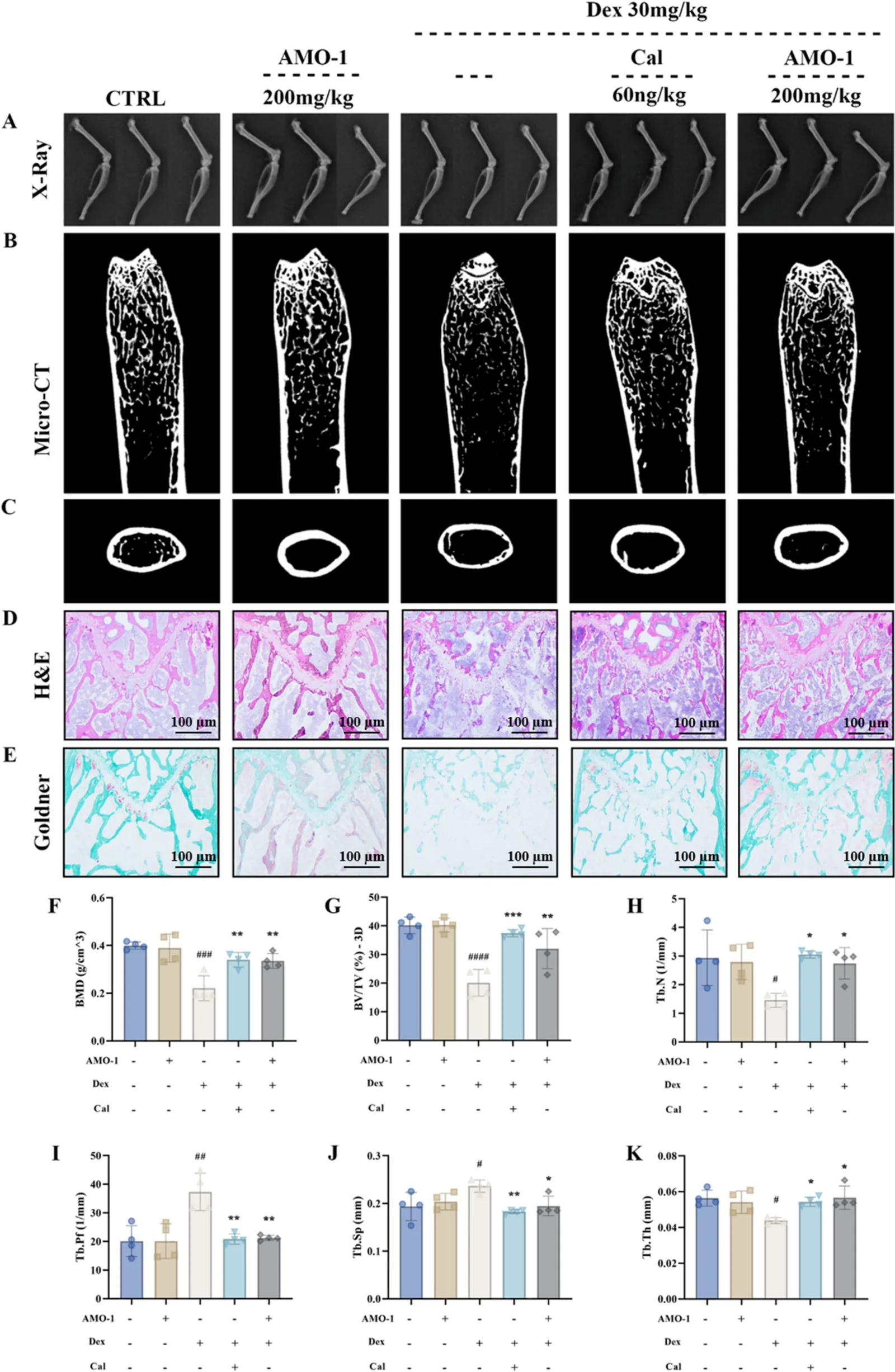
**A** X-ray images depicting the femur and tibia of mice (n = 3). **B** The coronal section images and **C** three-dimensional reconstructed visuals of the cortical bone. **D** Staining with H&E and **E** Goldner of the femur (n = 3, 100 × , scale bar: 100 μm). Quantitative assessments of (**F**) BMD, **G** BV/TV, **H** Tb.N, **I** Tb.Pf, **J** Tb.Sp, and **K** Tb.Th in the femur (n = 4). ^#^*P* < 0.05, ^##^*P* < 0.01, ^###^*P* < 0.001, and ^####^*P* < 0.0001 vs. CTRL mice; *P < 0.05, **P < 0.01, ***P < 0.001 vs. vehicle-treated OP mice

Subsequently, we investigated the microstructure of both the femur and tibia in different groups of mice using X-ray and micro-CT analyses. The results from the X-ray analysis indicated that the femur and tibia in the vehicle-treated OP mice exhibited diminished radiation absorption and a reduced cortical bone thickness relative to the CTRL group. Notably, the administration of AMO-1 led to a significant reversal of the pathological changes observed in the study (Fig. 6A). Furthermore, the micro-CT analysis demonstrated that the trabecular bones in the femur of the vehicle-treated OP mice were not only shorter but also exhibited reduced thickness compared to those in the CTRL group, characterized by loosely arranged and poorly interconnected reticular structures. Conversely, AMO-1 treatment led to a notable improvement in trabecular bone density and a restoration of the reticular architecture, consistent with the observations made in the X-ray analysis (Fig. 6B, C). In addition, when the vehicle-treated OP mice were evaluated against the CTRL group, a notable reduction in BMD, BV/TV, Tb.N, and Tb.Th was observed, along with a marked increase in Tb.Sp and Tb.Pf. Remarkably, administering AMO-1 at a dosage of 200 mg/kg led to a considerable rise in the measurements of BMD, BV/TV, Tb.N, and Tb.Th relative to the OP group, while also significantly reducing the values for Tb.Sp and Tb.Pf (Fig. 6F–K). Together, these results indicate that AMO-1 effectively mitigated DEX-induced bone loss. The restoration of trabecular structure following AMO-1 treatment was further validated by H&E staining (Fig. 6D) and Goldner staining (Fig. 6E). Furthermore, the outcomes noted in the vehicle-treated OP mice treated with Cal were similar to the results observed with AMO-1. Additionally, it was clear that the administration of AMO-1 by itself did not lead to any changes in bone mass among healthy mice (Fig. 6).

### AMO-1 promoted osteogenic differentiation in OP mice

**Fig. 7:**
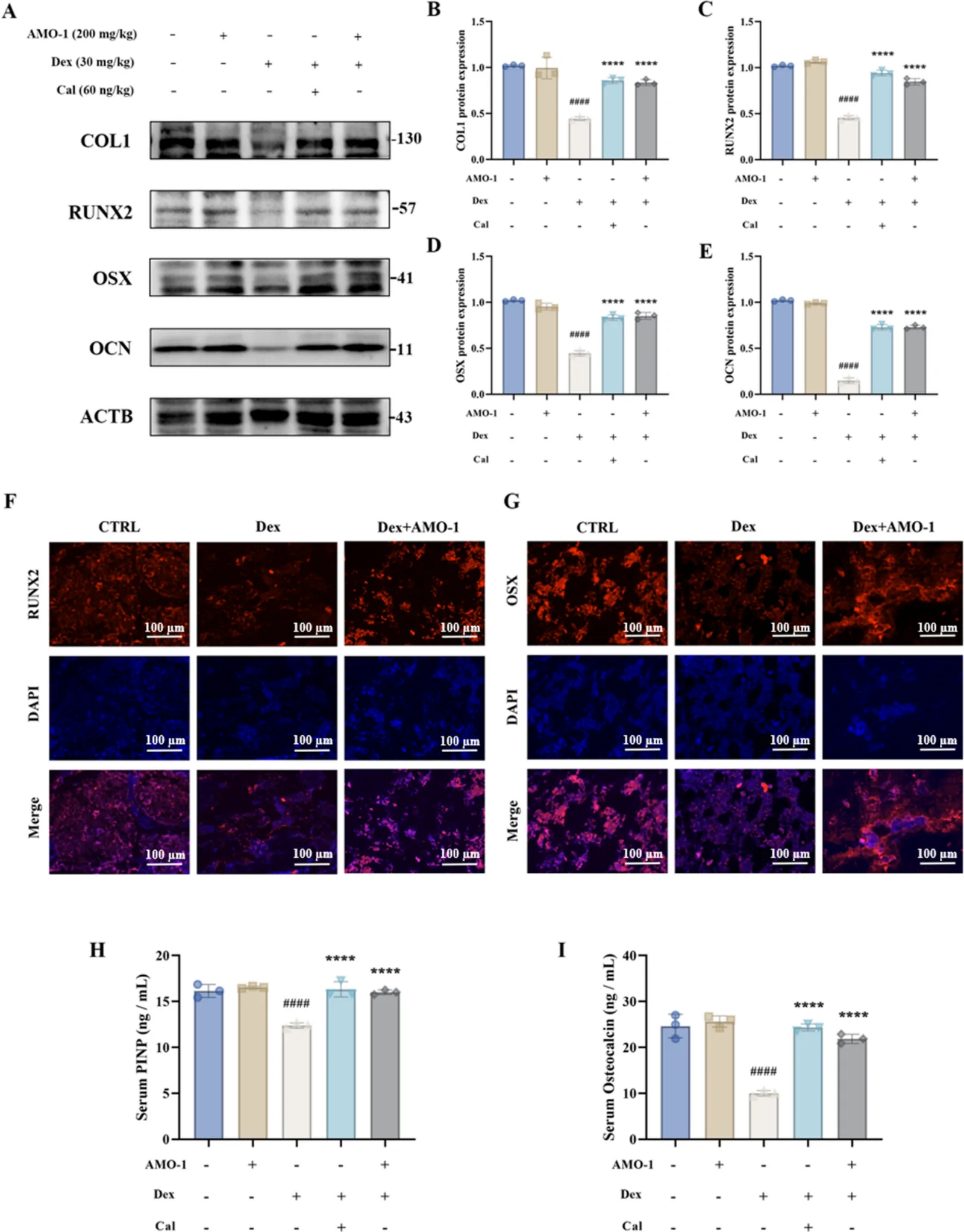
**A** Analysis via Western blot of protein expressions for RUNX2, OSX, OCN, and COL1 in the femurs of DEX-induced OP mice. **B** Measurements of RUNX2. **C** Measurements of OSX. **D** Measurements of OCN. **E** Measurements of COL1. Immunofluorescence staining images depicting femurs from DEX-induced OP mice with anti-RUNX2 (**F**) and anti-OSX (**G**) (100 × , scale bar: 100 μm). Serum concentrations of PINP (**H**) and osteocalcin (**I**). ^#^*P* < 0.05, ^##^*P* < 0.01, ^###^*P* < 0.001, and ^####^*P* < 0.0001 vs. CTRL mice; *P < 0.05, **P < 0.01, ***P < 0.001 vs. vehicle-treated OP mice, n = 3

Western blot analyses revealed a significant decrease in the levels of osteoblast activity markers, including RUNX2, OSX, OCN, and COL1, in the femurs of vehicle-treated OP mice in comparison to the CTRL group. Conversely, both AMO-1 and Cal treatments resulted in a marked increase in the levels of RUNX2, OSX, OCN, and COL1 when compared to the vehicle-treated group (Fig. 7A–E). It is crucial to highlight that AMO-1 alone did not result in a marked effect on the levels of osteogenesis-related proteins in healthy mice. Overall, these results indicate that AMO-1 may alleviate DEX-induced OP by facilitating osteogenic differentiation.

### Immunofluorescence assay

To further assess the impact of AMO-1 on the process of osteogenic differentiation, an immunofluorescence assay was performed. The findings, illustrated in Fig. 7F, G, reveal that the vehicle-treated group displayed a significant reduction in both the quantity of fluorescent bodies and the intensity of fluorescence relative to the CTRL group. In contrast, the administration of AMO-1 led to a considerable increase in both the quantity of fluorescent bodies and the fluorescence intensity when compared to the vehicle-treated group. These findings strongly suggest that AMO-1 significantly contributes to promoting osteogenic differentiation, which aligns with the results derived from the Western blot analysis.

### AMO-1 increased the serum levels of osteocalcin and PINP in OP mice

Serum levels of osteocalcin and PINP are frequently employed as biomarkers for bone formation in order to evaluate the anti-OP effects of different therapeutic agents. As demonstrated in Fig. 7H and I, the vehicle-treated group showed a marked decrease in serum levels of both PINP and osteocalcin relative to the CTRL group. Conversely, the administration of AMO-1 significantly increased the serum levels of both PINP and osteocalcin. Furthermore, the impact of Cal on the serum levels of PINP and osteocalcin in OP mice closely resembled that of AMO-1; however, when administered alone, AMO-1 did not alter the serum levels of these markers. These findings suggest that AMO-1 could alleviate DEX-induced OP via the promotion of bone formation.

### *AMO-1 promoted osteogenesis *via* PI3K/AKT/HIF-1α pathway based on proteomics analysis*

**Fig. 8:**
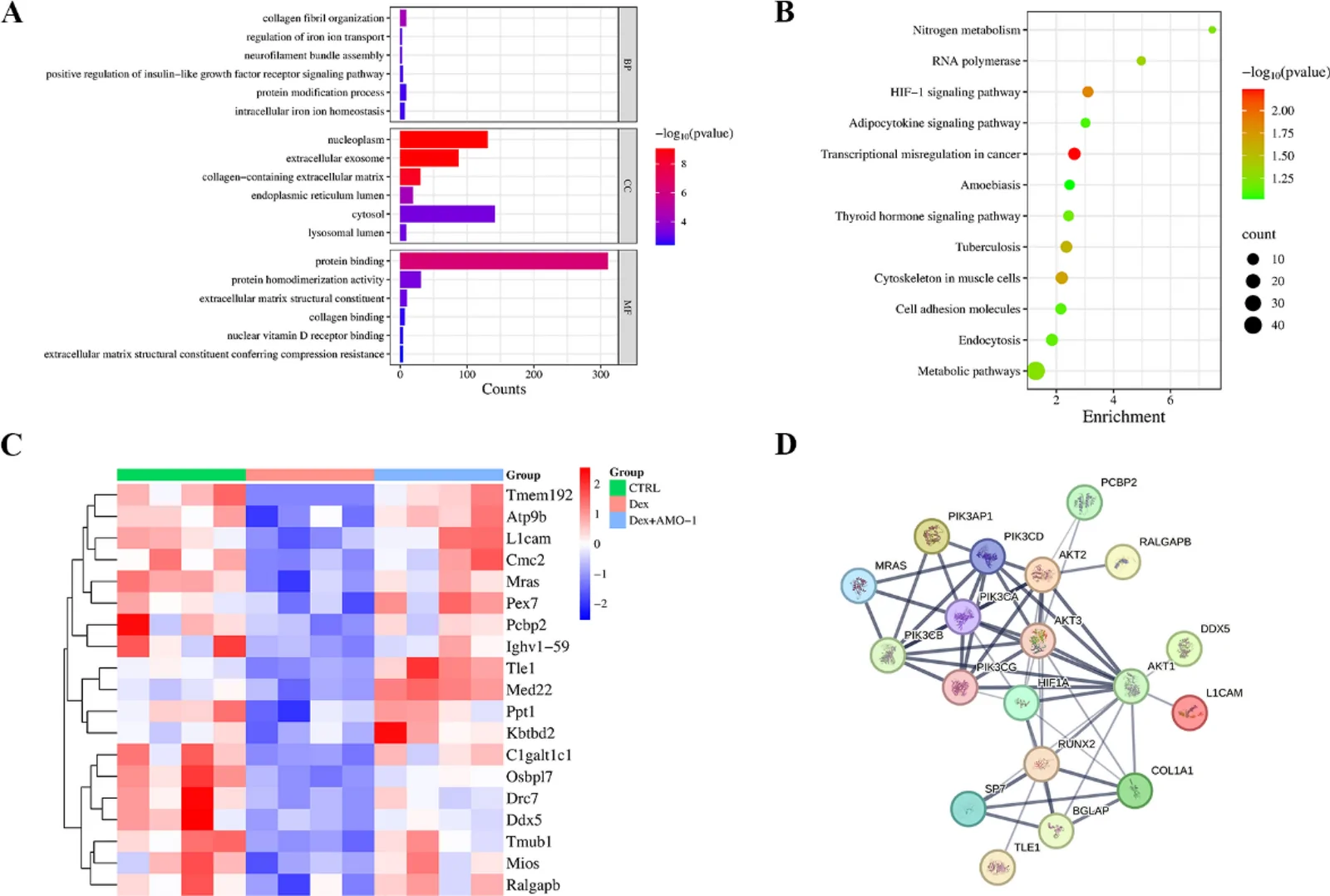
Analysis of proteomics conducted on femoral tissues from OP mice. **A** GO analysis and **B** KEGG analysis. **C** Heatmap analysis. **D** STRING analysis

**Fig. 9:**
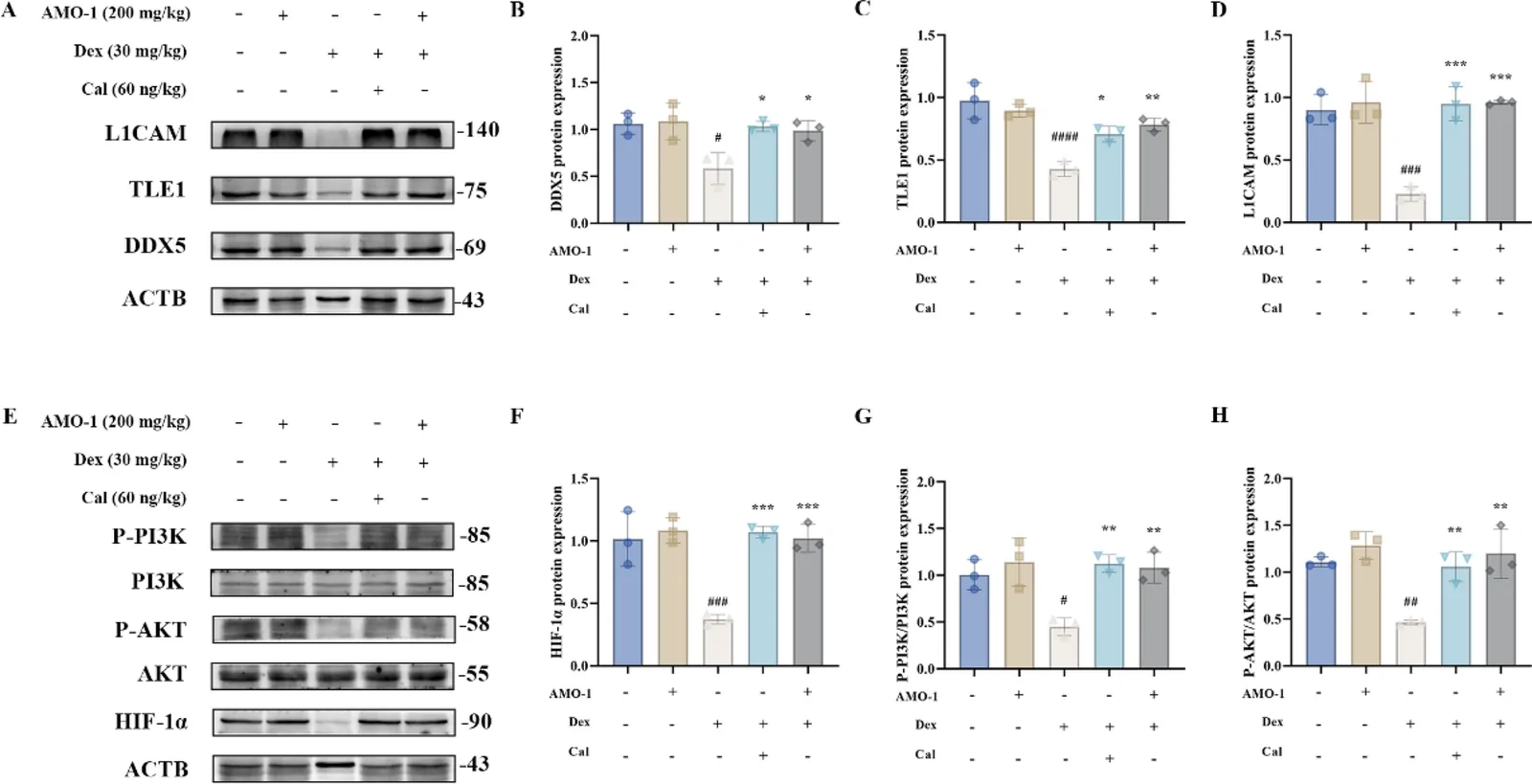
**A** Analysis via Western blotting of the levels of DDX5, TLE1, and L1CAM proteins in the femurs specimens. **B** Measurement of DDX5. **C** Measurement of TLE1. **D** Measurement of L1CAM. **E** Analysis via Western blotting of the expression levels of P-PI3K, PI3K, P-AKT, AKT, and HIF-1α proteins in the femurs specimens. **F** Measurement of HIF-1α. **G** Measurement of P-PI3K, normalized to PI3K. (H) Measurement of P-AKT, normalized to AKT. ^#^*P* < 0.05, ^##^*P* < 0.01, ^###^*P* < 0.001, and ^####^*P* < 0.0001 vs. CTRL mice; *P < 0.05, **P < 0.01, ***P < 0.001 vs. vehicle-treated OP mice, n = 3

In order to explore the possible mechanisms through which AMO-1 delivers its anti-OP effects, we performed a 4D label-free proteomics analysis comparing protein expression levels among the CTRL, vehicle-treated, and AMO-1-treated groups. The results illustrated in Fig. 8A indicate that the GO biological process (BP) terms predominantly pertain to collagen fibril organization, protein modification process, and intracellular iron ion homeostasis. Meanwhile, the GO cellular component (CC) terms were chiefly associated with nucleoplasm, extracellular exosome, and cytosol. Additionally, the GO molecular function (MF) terms were primarily enriched in protein homodimerization activity, extracellular matrix structural constituent, and protein binding. Furthermore, the KEGG analysis revealed that the DEPs are significantly enriched in the HIF-1α signaling pathway, metabolic pathway, and transcriptional misregulation in cancer (Fig. 8B). The role of the HIF-1α signaling pathway is vital in the development of OP. Importantly, HIF-1α facilitates fracture healing through the regulation of vital markers associated with osteogenic differentiation, including RUNX2, OSX, and COL1 [17]. In addition, the PI3K/AKT pathway is essential for the transduction of HIF-1α signals, with PI3K activation resulting in AKT phosphorylation, which in turn influences the downstream expression of HIF-1*α*. As illustrated in Fig. 8C and Table S2, a decrease in 19 different proteins, such as DEAD-Box Helicase 5 (DDX5), transducin-like enhancer of split 1 (TLE1), and L1 cell adhesion molecule (L1CAM), was noted in the vehicle-treated group, while treatment with AMO-1 significantly reversed these changes. Subsequently, we performed STRING analysis on the proteins influenced by AMO-1, along with indicators of osteogenic differentiation and the marker factors associated with the PI3K/AKT/HIF-1α signaling pathway. The findings revealed a significant correlation between these proteins (Fig. 8D). Additionally, consistent with the results of proteomics analysis, Western blot analysis demonstrated that the administration of AMO-1 markedly increased the expression levels of DDX5, TLE1, and L1CAM within the femoral tissues of DEX-induced OP mice (Fig. 9A–D).

In light of the analyses performed using KEGG and STRING databases, we undertook a more detailed investigation into the potential mechanisms by which AMO-1 alleviates DEX-induced OP. Our focus was particularly on the PI3K/AKT/HIF-1α signaling pathway, for which we employed a Western blot assay to examine specific protein levels. The findings, as demonstrated in Fig. 9G, H, reveal that the phosphorylated levels of both PI3K and AKT, denoted as P-PI3K/PI3K and P-AKT/AKT, were markedly reduced in the vehicle-treated group relative to the CTRL group. Conversely, the administration of AMO-1 resulted in a notable increase in the levels of P-PI3K/PI3K and P-AKT/AKT relative to the vehicle-treated group. Furthermore, treatment with AMO-1 effectively reversed the decrease in HIF-1α expression (Fig. 9F). It is important to note that AMO-1 by itself did not lead to any notable changes in the protein concentrations of HIF-1*α*, P-PI3K/PI3K, and P-AKT/AKT in healthy mice, as depicted in Fig. 9. Overall, these results suggest that AMO-1 could offer protective benefits against OP via the stimulation of the PI3K/AKT/HIF-1α signaling pathway.

## Discussion

In this study, a neutral oligosaccharide AMO‑1 with a molecular weight of 666 Da was successfully isolated and purified from *A. marina* fruits. Its chemical structure was elucidated as α-D-Gal*p*-(1 → 6)-α-D-Gal*p*-(1 → 6)-α-D-Glc*p*-(1 → 2)-β-D-Fru*f*. Both in vitro and in vivo experiments verified its anti‑OP activity. Numerous studies have demonstrated that the structural features of natural saccharides are closely associated with their biological activities [12]. For example, MOW50-1, a fructose- and glucose-containing polysaccharide from *Morinda officinalis*, increases ALP activity to promote osteogenic differentiation of MC3T3-E1 cells [21]. PSP50-2–2, a heteropolysaccharide rich in Gal, Glc and Fru isolated from *Polygonatum sibiricum*, markedly facilitates osteoblast differentiation and mineralization [22]. Similarly, ABW90-1, an oligosaccharide obtained from *Achyranthes bidentata*, is mainly composed of Fru and Glc. It consists of (2 → 6)-linked-*β*‑D‑fructofuranosyl (Fru*f*) and (2 → 1)-linked-*β*‑D‑Fru*f* residues with Glc and Fru as terminal groups, and can effectively promote the proliferation and differentiation of primary osteoblasts [23]. Notably, both AMO‑1 and ABW90‑1 share a core Fru‑Glc skeleton, which is considered responsible for their anti‑OP effects. Nevertheless, AMO‑1 contains extra Gal residues and exhibits distinct glycosidic linkage patterns, thus forming a novel structural type that has not been reported among anti-OP oligosaccharides. The conserved Fru‑Glc fragments may serve as key functional motifs for osteogenesis, and the unique structural features of AMO-1 provide new perspectives for further investigating the structure–activity relationships of anti-OP saccharides.

Long-term and high-dose glucocorticoid treatment can inhibit osteoblast differentiation, maturation, and bone formation, leading to OP. Therefore, we investigated the in vivo anti-OP activity of AMO-1 using a DEX-induced OP mouse model. X-ray and micro-CT analyses demonstrated that the administration of AMO-1 significantly enhanced trabecular bone density, restored the femoral reticular structure, and improved bone morphometric parameters in DEX-induced OP mice. These findings were further corroborated by H&E staining and Goldner's staining. Additionally, Western blot and immunofluorescence experiments showed that the expression levels of osteoblast activity markers such as RUNX2, OSX, OCN, and COL1 were significantly reduced in the femoral tissues of DEX-induced OP mice, and these reductions were effectively reversed following AMO-1 intervention. These results were similar to previous studies demonstrating the anti-OP effects of natural saccharides derived from *Chroogomphus rutilus* [24], *Codonopsis pilosula* [25], and *Dendrobium offificinale* [26]. However, this study only preliminarily explored the anti-OP pharmacological effects of AMO-1 using a single effective dose. Further multi-dose animal experiments will be carried out in follow-up studies to clarify its dose–effect relationship and screen out the optimal therapeutic dose of AMO-1.

Proteomic analysis revealed that AMO-1 treatment significantly upregulated the expression levels of DDX5, TLE1, and L1CAM in the femoral tissues of DEX-induced OP mice, which was further confirmed by Western blot experiments. Additionally, STRING analysis demonstrated a strong correlation among these DEPs, osteogenesis-related proteins, and key markers of the PI3K/AKT/HIF-1α signaling pathway. DDX5, a multifunctional DEAD-box protein, plays a crucial role in RNA metabolism [27]. A prior investigation has shown that DDX5 is intricately linked to the differentiation and maturation of osteoblasts [28]. TLE1, a significant member of the TLE protein family, has been demonstrated to influence the differentiation of bone marrow mesenchymal stem cells (BMSCs) into adipogenic and osteogenic lineages [29]. Furthermore, TLE1 can impact various cellular biological functions by modulating the PI3K/AKT signaling pathway [30]. L1CAM, a molecule crucial for cell adhesion and a member of the immunoglobulin superfamily, was initially recognized for its vital role in the development of the nervous system [31]. Evidence suggests that miR-214-3p, a small endogenous non-coding RNA, can modulate endothelial cell function by targeting L1CAM, thereby influencing angiogenesis and vascular architecture in the bone microenvironment while also accelerating bone degenerative processes [32]. Collectively, our findings indicate that proteins such as DDX5, TLE1, and L1CAM are closely associated with OP, aligning with previous studies. The results of our study may provide novel targets for the clinical diagnosis and treatment of OP.

The PI3K/AKT signaling pathway is a critical regulator of osteoblast proliferation, differentiation, and bone formation [33]. In this pathway, PI3K acts as a lipid kinase that generates phosphatidylinositol-3,4,5-trisphosphate (PIP3), which subsequently activates AKT through phosphatidylinositol-dependent kinase 1 (PDK1) [34]. AKT serves as the primary signaling molecule activated by PI3K, regulating several downstream targets, including glycogen synthase kinase-3β (GSK-3β), the mammalian target of rapamycin (mTOR), and HIF [35]. A recent study has shown that the activation of the PI3K/AKT pathway can enhance bone formation and inhibit bone loss associated with glucocorticoid-induced OP [11]. Notably, HIF-1α, as a downstream target of the PI3K/AKT signaling pathway, plays a crucial role in the progression of several diseases, including osteoarthritis, osteonecrosis, and OP, primarily due to its influence on the proliferation and osteogenic differentiation of BMSCs [36]. Recently, it has been demonstrated that the activation of the HIF signaling pathway enhances the expression level of the downstream gene VEGF, thereby facilitating bone regeneration in OP [37]. Furthermore, HIF-1α promoted glycolysis within the bone marrow microenvironment by up-regulating the key enzyme PDK1, which subsequently induced osteoblast differentiation and stimulated bone formation [38]. Additionally, increased HIF-1α expression promoted angiogenesis and osteogenic differentiation of BMSCs, further promoting osteogenesis. In this study, based on the results of KEGG and STRING analyses, we hypothesized that AMO-1 may ameliorate DEX-induced OP through the PI3K/AKT/HIF-1α signaling pathway. Western blot analysis revealed that AMO-1 treatment significantly upregulated the expression levels of P-PI3K/PI3K, P-AKT/AKT, and HIF-1α. Thus, the activation of the PI3K/AKT/HIF-1α signaling pathway represents the key mechanism by which AMO-1 displays its anti-OP properties. However, this study only preliminarily confirmed that AMO-1 exerts its anti-OP effects through the PI3K/AKT/HIF-1α pathway. Due to the failure to perform pathway inhibition experiments using specific pathway inhibitors or siRNA-mediated gene silencing technology, the reverse verification of the PI3K/AKT/HIF-1α signaling axis is insufficient, which is one of the main limitations of this study. Therefore, future studies may adopt specific pathway inhibitors and siRNA interference technology to conduct loss-of-function experiments, so as to further verify the specific regulatory effect of AMO-1 on the PI3K/AKT/HIF-1α pathway, and deeply clarify the specific regulatory mechanisms of its upstream and downstream, thereby improving the research on the molecular mechanism of AMO-1 in anti-OP.

In conclusion, a neutral tetrasaccharide identified as AMO-1 was isolated from the fruits of *A. marina* utilizing Sephacryl S-200 and DEAE-cellulose 52 column chromatography. The exact structure of AMO-1 was determined to be α-D-Gal*p*-(1 → 6)-α-D-Gal*p*-(1 → 6)-α-D-Glc*p*-(1 → 2)-β-D-Fru*f*, according to analyses including monosaccharide composition, methylation studies, UPLC-ESI–MS/MS, and NMR spectroscopy. Furthermore, AMO-1 exhibited anti-OP effects in both vitro and vivo settings. AMO-1 significantly enhanced the expression of proteins crucial for osteogenesis, which led to an observable promotion of osteogenic differentiation and mineralization in MC3T3-E1 cells. Furthermore, in a DEX-induced OP mouse model, treatment with AMO-1 significantly reduced bone loss, increased the levels of osteogenic factors, and increased serum levels of osteocalcin and PINP. Additionally, proteomic analysis and Western blot results indicated that AMO-1 may facilitate osteogenesis through the activation of the PI3K/AKT/HIF-1α signaling pathway. Overall, our findings suggest that AMO-1 has the potential for development as a functional ingredient in the prevention and management of OP.

## Materials and methods

### Materials and reagents

The fruits of *A. marina* were gathered from Zhanjiang City in Guangdong, China. Monosaccharide standards were sourced from Sigma-Aldrich Co. (St. Louis, USA). Alizarin red S (ARS), an alkaline phosphatase (ALP) assay kit, a BCA protein assay kit, and the cell counting kit-8 (CCK-8) were acquired from Beyotime Biotech Co., Ltd (Shanghai, China). Dulbecco’s modified eagle medium (DMEM) along with additional cell culture components were sourced from Gibco BRL Co., Ltd (California, USA). Antibodies were procured from Signalway Antibody Co. Ltd. (Maryland, America). All other chemicals and reagents were commercially available and of analytical grade.

### Preparation of AMO-1

The preparation of AMO-1 was conducted in accordance with previously established methods [39]. Briefly, the fruits of *A. marina* were subjected to extraction three times with deionized water at a mass-to-volume ratio of 1:30 (w/v, kg/L) and maintained at a temperature of 80°C for 2 h. Afterward, the resulting extracts were combined, concentrated, and then added with four volumes of 95% ethanol, and allowed to settle overnight for precipitation. The obtained precipitate was then separated through centrifugation, deproteinized, and freeze-dried to yield crude oligosaccharides (AMO). Subsequently, AMO was further refined using DEAE-52-cellulose and Sephacryl S-200 columns, leading to the isolation of the purified oligosaccharide designated as AMO-1.

### General properties of AMO-1

Quantification of the total contents of carbohydrate, protein, and uronic acid in AMO-1 was performed utilizing the phenol‑sulfuric acid method, Bradford method, and *m*-hydroxydiphenyl method respectively. A UV spectrophotometer was utilized to evaluate the presence of nucleic acids and proteins in AMO-1. Additionally, a Fourier transform infrared spectroscopy (FT-IR) spectrometer was utilized to acquire the FT-IR spectrum for AMO-1.

### Homogeneity and molecular weight determination of AMO-1

The assessment of the homogeneity and molecular weight of AMO-1 was performed using high-performance gel permeation chromatography (HPGPC). Moreover, the molecular weight of AMO-1 was established through UPLC-ESI–MS/MS, following the procedures described in an earlier study [40].

### Monosaccharide composition analysis of AMO-1

Fructose (Fru) is prone to degradation if subjected to elevated temperatures over prolonged durations, which makes accurate quantification challenging. Moreover, in the methylation derivatization process, the carbonyl group at the 2-position of Fru undergoes reduction, leading to the formation of glucose (Glc) and mannitol, which are then transformed into sugar alcohol acetate ester derivatives. To address these challenges, we utilized two different hydrolysis methods to investigate the monosaccharide composition of AMO-1 [41].

In the first method, hydrolysis of 5 mg of AMO-1 was carried out using 3 M trifluoroacetic acid (TFA) at 120°C for 3 h. The alternative method involved performing hydrolysis with 2 M TFA at 60°C for 30 min. Following this, the resulting hydrolysate was subjected to centrifugation at 12,000 rpm for 5 min, and the supernatant was examined with an ICS 5000 ion chromatography system. The monosaccharide components contained in AMO-1 were identified and quantified by comparing their peak areas and retention times against those of established monosaccharide standards.

### Methylation analysis of AMO-1

The examination of AMO-1 methylation was performed based on our earlier outlined procedure, with slight modifications [39]. In summary, methylated products were isolated utilizing chloroform and then analyzed using FT-IR spectroscopy. The lack of a hydroxyl absorption peak confirmed that AMO-1 had been fully methylated. Subsequently, the product underwent hydrolysis with 2 M TFA at 120°C for 90 min. This was followed by an 8-h reaction with NaBH_4_, and the final step involved acetylation with acetic anhydride and pyridine at 100°C for 1 h. Lastly, partially methylated alditol acetates (PMAAs) were assessed through gas chromatography-mass spectrometry (GC–MS) to identify the glycosidic linkages present in AMO-1.

### NMR analysis of AMO-1

A solution containing 40 mg of AMO-1 was dissolved in D_2_O. The NMR spectra for AMO-1, including both 1D and 2D, were obtained using a Bruker AV-600 spectrometer from Bruker.

### Scanning electron microscopy (SEM) analysis of AMO-1

The sample holder was affixed with dried AMO-1 using conductive adhesive, followed by the application of gold powder spray. Following this, a scanning electron microscope was employed to analyze the surface morphology of AMO-1. SEM images were captured at magnifications of 500 × and 2000 × , respectively.

### Cell viability assay

MC3T3-E1 cells (CL-0378, Subclone 14) from Pricella Biotechnology in Wuhan, China, are bone-forming cells derived from mice. These cells were cultured in DMEM in a 5% CO_2_ incubator set at 37℃. They were plated in 96-well plates and exposed to different concentrations of AMO-1 (0, 12.5, 25, and 50 μg/mL) for 24 h at 37℃. To assess cell viability, CCK-8 assays were conducted according to the guidelines provided by the manufacturer.

### ALP staining and ARS staining assays

MC3T3-E1 cells were seeded in 24-well plates and incubated for 24 h. Subsequently, the culture medium was replaced with an osteogenic differentiation medium that included varying concentrations of AMO-1, specifically 0, 12.5, 25, and 50 μg/mL. Following a treatment duration of either 7 or 14 days, the MC3T3-E1 cells were fixed using a 4% paraformaldehyde (PFA) solution for a period of 30 min. After fixation, the cells were subjected to either an ALP staining solution or an ARS staining solution, which was also applied for an additional 30 min. Throughout this entire experimental protocol, precautions were taken to shield the cells from light exposure, in strict accordance with the manufacturer's guidelines. Subsequently, the stained cells were observed under a stereomicroscope, and the images captured were processed and analyzed with the assistance of ImageJ software.

### Animals and treatments

All procedures involving animals were performed following the guidelines set forth by the National Research Council for the Care and Use of Laboratory Animals, while also complying with the ARRIVE standards and regulations from the U.S. National Institutes of Health (NIH). The Animal Experimentation Ethics Committee at Guangdong Medical University approved all experimental protocols (Approval No. GDMU-2024-000016). Forty male C57BL/6 mice were sourced from SiPeiFu (Beijing) Biotechnology Co., Ltd. The mice were maintained in a pathogen-free environment with a 12-h light/dark cycle, and they had continuous access to sterilized food and water. Following a week-long adjustment period, the mice were randomly assigned to five groups (n = 8 for each group). In the experimental design, the normal control (CTRL) group and AMO-1 treatment group were administered double distilled (D.D.) water and AMO-1, respectively, via intragastric delivery at a specified dosage of 200 mg/kg. Both groups also received daily intraperitoneal injections of normal saline to maintain consistency in the treatment regimen. On the other hand, the groups treated with the vehicle, AMO-1, and positive control drug (calcitriol, Cal; 60 ng/kg) received intragastric administration of D.D. water, AMO-1 (200 mg/kg), and calcitriol, followed by daily intraperitoneal injections of dexamethasone (DEX; 30 mg/kg). The mice were observed on a daily basis, and their body weights were documented weekly. Following 63 days of consistent administration, the mice were euthanized, and the femur, tibia, as well as several organs including the liver, spleen, and kidneys, were gathered for additional analysis.

### X-ray and micro-computed tomography (micro-CT) analysis

The tibia and femur were placed in a 4% PFA solution prior to evaluation using an X-ray machine and a SkyScan 1172 micro-CT system. Data analysis was conducted with the help of software (CTAn), which assessed parameters associated with the trabecular bone in the distal femoral metaphysis and the cortical bone in the diaphysis. The region of interest (ROI) included the entire trabecular bone of the femoral head. Additionally, various morphometric parameters were evaluated, such as bone mineral density (BMD), bone volume fraction (BV/TV), trabecular number (Tb. N), trabecular thickness (Tb. Th), trabecular pattern factor (Tb. Pf), and trabecular spacing (Tb. Sp).

### Histopathological analysis

The organ tissues that were collected, which included the liver, spleen, and kidney, were prefixed in a 4% PFA solution for 24 h. Meanwhile, the femur tissues were subjected to decalcification in a specific solution for 7 days. Subsequently, dehydration of the tissues was performed using ethanol, followed by embedding in paraffin. Afterward, the paraffin-embedded tissue sections were stained with hematoxylin and eosin (H&E). Additionally, the femur tissues were treated with Goldner staining following the manufacturer's instructions (GP1053; Servicebio; Wuhan, China).

### Immunofluorescence assay

Immunofluorescence staining was performed on histological slices of femur tissues. Initially, the paraffin sections were heated at 65°C for 2 h, followed by dewaxing with xylene and hydration through various ethanol concentrations. After rinsing with double-distilled water, the specimens were subjected to an antigen retrieval solution at 100°C for 20 min. Following this, the sections were incubated in a blocking solution with 5% bovine serum albumin (BSA) at 37°C for 30 min, and they were then treated overnight at 4°C with primary antibodies (OSX and RUNX2). Afterward, secondary antibodies were used for 2 h at 25°C, and the sections were gradually sealed using a sealing medium that contained DAPI. Stained sections were observed with a spinning disk confocal microscope.

### Evaluation of serum markers of bone formation

Blood samples were subjected to centrifugation at 4000 rpm for 20 min at 4℃ in order to separate the serum. The evaluation of serum levels for osteocalcin and procollagen Type I N-terminal propeptide (PINP), both of which serve as key indicators of bone formation, was conducted utilizing the osteocalcin ELISA Kit (RX202908M; RUIXIN Biotech; Quanzhou, China) and the PINP ELISA Kit (E-EL-M0233; Elabscience; Wuhan, China), following the protocols outlined by the manufacturers.

### Proteomics analysis

Samples of femur tissue from the CTRL, vehicle-treated, and AMO-1-treated mouse groups were ground using liquid nitrogen. A suitable volume of protein lysate was subsequently added, followed by centrifugation to isolate the protein supernatant. The protein level was assessed via a BCA protein assay kit. Next, SDS-PAGE electrophoresis was conducted to determine if the quality of the samples was adequate for further experiments. Afterward, a reductive alkylation reaction was performed on the validated protein samples. Protein digestion was performed using trypsin as outlined in the filter-aided sample preparation (FASP) technique. Subsequent to this, the resultant peptides underwent analysis via LC–MS/MS, with the acquired raw mass spectrometry data being aligned to a database to facilitate the identification and quantification of the peptides. Proteins were categorized as differentially expressed proteins (DEPs) when they displayed a *p*-value < 0.05 and a fold change (FC) > 1.2 or < 0.83. Based on the differences in protein expression identified among the CTRL, vehicle-treated, and AMO-1-treated groups, alongside bioinformatics evaluations such as gene ontology (GO) enrichment and kyoto encyclopedia of genes and genomes (KEGG) enrichment analyses, we explored the possible mechanisms through which AMO-1 could aid in the prevention and management of OP.

### Western blot analysis

Cells and femoral tissues underwent lysis, and then the protein concentration in the obtained supernatants was measured using a BCA protein assay kit. Following quantification to 40 μg, the samples were subjected to separation by 10% SDS-PAGE and then transferred onto polyvinylidene fluoride membranes. After a 30-min incubation with a quick closure solution, the membranes were exposed to primary antibodies overnight at 4℃. This was followed by a 1-h incubation with secondary antibodies at 37℃. Finally, protein visualization was conducted with the ECL detection system, and band intensity was assessed using ImageJ software.

### Statistical analysis

The results are shown as the mean ± standard error of the mean (S.E.M.). Statistical assessments were conducted utilizing GraphPad Prism 8.0 software. Statistical differences were assessed through Student’s *t*-test or one-way analysis of variance (ANOVA). A value of *P* < 0.05 was deemed statistically significant.

## Supplementary Information

Supplementary Material 1.

## Data Availability

Data will be made available on request.

## References

[1] Harvey N, Dennison E, Cooper C. Osteoporosis: impact on health and economics. Nat Rev Rheumatol. 2010;6:99–105. 10.1038/nrrheum.2009.260. 20125177

[2] Zhou SY, Huang GL, Cheng GY. Synthesis and biological activities of drugs for the treatment of osteoporosis. Eur J Med Chem. 2020;197:112313. 10.1016/j.ejmech.2020.112313. 32335412

[3] Lu JJ, Hu DS, Ma C, Shuai B. Advances in our understanding of the mechanism of action of drugs (including traditional Chinese medicines) for the intervention and treatment of osteoporosis. Front Pharmacol. 2022;13:938447. 10.3389/fphar.2022.938447. PMC9237325 35774616

[4] Song JY, Zhang YF, Zhu YF, Jin XH, Li LZ, Wang CY, et al. Structural characterization and anti-osteoporosis effects of polysaccharide purified from *Eucommia ulmoides* Oliver cortex based on its modulation on bone metabolism. Carbohydr Polym. 2023;306:120601. 10.1016/j.carbpol.2023.120601. 36746570

[5] Eldohaji LM, Hamoda AM, Hamdy R, Soliman SS. *Avicennia marina* a natural reservoir of phytopharmaceuticals: Curative power and platform of medicines. J Ethnopharmacol. 2020;263:113179. 10.1016/j.jep.2020.113179. 32768642

[6] Zhou P, Hu HH, Wu X, Feng ZH, Li XY, Tavakoli S, et al. Botany, traditional uses, phytochemistry, pharmacological activities, and toxicity of the mangrove plant *Avicennia marina*: a comprehensive review. Phytochem Rev. 2025;24:5533–68. 10.1007/s11101-025-10080-2.

[7] Fang XB, Chen XE. Structure elucidation and immunological activity of a novel pectic polysaccharide from the stems of *Avicennia marina*. Eur Food Res Technol. 2013;236:243–8. 10.1007/s00217-012-1877-6.

[8] Yuan QX, Lv KL, Huang JW, Sun SJ, Fang ZY, Tan HJ, et al. Simulated digestion, dynamic changes during fecal fermentation and effects on gut microbiota of *Avicennia marina* (Forssk.) Vierh. fruit non-starch polysaccharides. Food Chem X. 2022;16:100475. 10.1016/j.fochx.2022.100475. PMC9574768 36263243

[9] An J, Yang H, Zhang Q, Liu CC, Zhao JJ, Zhang LL, et al. Natural products for treatment of osteoporosis: the effects and mechanisms on promoting osteoblast-mediated bone formation. Life Sci. 2016;147:46–58. 10.1016/j.lfs.2016.01.024. 26796578

[10] Ebeling PR, Nguyen HH, Aleksova J, Vincent AJ, Wong P, Milat F. Secondary osteoporosis. Endocr Rev. 2022;43:240–313. 10.1210/endrev/bnab028. 34476488

[11] Chen M, Zhou SQ, Sun K, Hu YJ, Wen HY, Ren GL. Andrographolide protects BMSCs from dexamethasone-induced cellular dysfunction and promotes bone formation via the PI3K/AKT pathway. J Funct Foods. 2023;105:105576. 10.1016/j.jff.2023.105576.

[12] Lei SS, Su J, Zhang Y, Huang XW, Wang XP, Huang MC, et al. Benefits and mechanisms of polysaccharides from Chinese medicinal herbs for anti-osteoporosis therapy: A review. Int J Biol Macromol. 2021;193:1996–2005. 10.1016/j.ijbiomac.2021.11.030. 34767882

[13] Zheng H, He B, Wu TX, Cai J, Wei JS. Extraction, purification and anti-osteoporotic activity of a polysaccharide from *Epimedium brevicornum* Maxim. in vitro. Int J Biol Macromol. 2020;156:1135–45. 10.1016/j.ijbiomac.2019.11.145. 31751710

[14] Sun X, Wei B, Peng ZH, Fu QL, Wang CJ, Zhen JC, et al. Protective effects of *Dipsacus asper* polysaccharide on osteoporosis *in vivo* by regulating RANKL/RANK/OPG/VEGF and PI3K/Akt/eNOS pathway. Int J Biol Macromol. 2019;129:579–87. 10.1016/j.ijbiomac.2019.02.022. 30735778

[15] Bie MM, Tang Y, Xia YX, Zhang Q, Tian YY, Cheng CA, et al. HIF-1α mediates osteoclast-induced disuse osteoporosis via cytoophidia in the femur of mice. Bone. 2023;168:116648. 10.1016/j.bone.2022.116648. 36563716

[16] Xu WN, Zheng HL, Yang RZ, Jiang LS, Jiang SD. HIF-1α regulates glucocorticoid-induced osteoporosis through PDK1/AKT/mTOR signaling pathway. Front Endocrinol. 2020;10:922. 10.3389/fendo.2019.00922. PMC6997475 32047474

[17] Zhang L, Jin L, Guo JL, Bao K, Hu JL, Zhang YZ, et al. Chronic intermittent hypobaric hypoxia enhances bone fracture healing. Front Endocrinol. 2021;11:582670. 10.3389/fendo.2020.582670. PMC7921462 33664707

[18] Wang TG, Wu ZN, Li MF, Cao BC, Li J, Jiang JH, et al. TCP80–1, a new levan-neoseries fructan from *Tupistra chinensis* Baker rhizomes alleviates ulcerative colitis induced by dextran sulfate sodium in Drosophila melanogaster model. Food Res Int. 2025;203:115860. 10.1016/j.foodres.2025.115860. 40022382

[19] Ye ZQ, Li TY, Qing DG, Sun Y, Chen HY, Yu Q, et al. Structural elucidation and osteogenic activity of a novel heteropolysaccharide from *Alhagi pseudalhagi*. Int J Biol Macromol. 2021;171:185–97. 10.1016/j.ijbiomac.2020.12.189. 33412197

[20] Liang J, Wang WF, Zhang Y, Chai YQ, Li YG, Jiang SL, et al. Fructooligosaccharides and fructans from *Platycodon grandiflorum*: structural characterization, lung-oriented guidance and targetability. Carbohydr Polym. 2024;323:121457. 10.1016/j.carbpol.2023.121457. 37940316

[21] Zhang DW, Zhang SJ, Jiang KM, Li TY, Yan CY. Bioassay-guided isolation and evaluation of anti-osteoporotic polysaccharides from *Morinda officinalis*. J Ethnopharmacol. 2020;261:113113. 10.1016/j.jep.2020.113113. 32668320

[22] Liu J, Li TY, Chen HY, Yu Q, Yan CY. Structural characterization and osteogenic activity *in vitro* of novel polysaccharides from the rhizome of *Polygonatum sibiricum*. Food Funct. 2021;12:6626–36. 10.1039/D1FO00938A. 34105561

[23] Wang CS, Zhang DW, Zhang ML, Jiao YK, Jiang KM, Yan CY. Structural characterization of a novel oligosaccharide from *Achyranthes bidentata* and its anti-osteoporosis activities. Ind Crops Prod. 2017;108:458–69. 10.1016/j.indcrop.2017.07.018.

[24] Zhou Y, Li LZ, Sun Z, Liu R, Zhu YF, Yi JZ, et al. Structural characterization and osteogenic differentiation-promoting activity of polysaccharide purified from *Chroogomphus rutilus*. Carbohydr Polym. 2024;328:121709. 10.1016/j.carbpol.2023.121709. 38220343

[25] Liu JJ, An JY, Jiang N, Yang K, Guan CH, Zhao N, et al. *Codonopsis pilosula* polysaccharides promote osteogenic differentiation and inhibit lipogenic differentiation of rat bone marrow stem cells by activating β-catenin. Chem Biol Interact. 2023;385:110721. 10.1016/j.cbi.2023.110721. 37739048

[26] Wang YJ, Jiang ZJ, Deng LH, Zhang GM, Xu X, Alonge E, et al. *Dendrobium offificinale* polysaccharides prevents glucocorticoids-induced osteoporosis by destabilizing KEAP1-NRF2 interaction. Int J Biol Macromol. 2023;253:126600. 10.1016/j.ijbiomac.2023.126600 37652317

[27] Legrand JMD, Chan AL, La HM, Rossello FJ, Änkö ML, Fuller-Pace FV, et al. DDX5 plays essential transcriptional and post-transcriptional roles in the maintenance and function of spermatogonia. Nat Commun. 2019;10:2278. 10.1038/s41467-019-09972-7. PMC6533336 31123254

[28] Jensen ED, Niu LL, Caretti G, Nicol SM, Teplyuk N, Stein GS, et al. p68 (Ddx5) interacts with Runx2 and regulates osteoblast differentiation. J Cell Biochem. 2008;103:1438–51. 10.1002/jcb.21526. 17960593

[29] Kokabu S, Lowery JW, Jimi E. Cell fate and differentiation of bone marrow mesenchymal stem cells. Stem Cells Int. 2016;2016:3753581. 10.1155/2016/3753581. PMC4889852 27298623

[30] Zhang K, Wang ZC, Sun HX, Long HM, Wang YJ. Esculentoside H reduces the PANoptosis and protects the blood-brain barrier after cerebral ischemia/reperfusion through the TLE1/PI3K/AKT signaling pathway. Exp Neurol. 2024;379:114850. 10.1016/j.expneurol.2024.114850. 38857750

[31] Kiefel H, Bondong S, Hazin J, Ridinger J, Schirmer U, Riedle S, et al. L1CAM: a major driver for tumor cell invasion and motility. Cell Adhes Migr. 2012;6:374–84. 10.4161/cam.20832. PMC3478260 22796939

[32] Guo Z, Li J, Tan JY, Sun SN, Yan Q, Qin H. Exosomal miR-214-3p from senescent osteoblasts accelerates endothelial cell senescence. J Orthop Surg Res. 2023;18:391. 10.1186/s13018-023-03859-6. PMC10227955 37248458

[33] Xi JC, Zang HY, Guo LX, Xue HB, Liu XD, Bai YB, et al. The PI3K/AKT cell signaling pathway is involved in regulation of osteoporosis. J Recept Signal Transduction. 2015;35:640–5. 10.3109/10799893.2015.1041647. 26390889

[34] Xiang YB, Yang YX, Liu J, Yang X. Functional role of MicroRNA/PI3K/AKT axis in osteosarcoma. Front Oncol. 2023;13:1219211. 10.3389/fonc.2023.1219211. PMC10315918 37404761

[35] Liu PX, Cheng HL, Roberts TM, Zhao JJ. Targeting the phosphoinositide 3-kinase pathway in cancer. Nat Rev Drug Discov. 2009;8:627–44. 10.1038/nrd2926. PMC3142564 19644473

[36] Chen W, Wu PF, Yu F, Luo GJ, Qing LM, Tang JY. HIF-1α regulates bone homeostasis and angiogenesis, participating in the occurrence of bone metabolic diseases. Cells. 2022;11:3552. 10.3390/cells11223552. PMC9688488 36428981

[37] Kusumbe AP, Ramasamy SK, Adams RH. Coupling of angiogenesis and osteogenesis by a specific vessel subtype in bone. Nature. 2014;507:323–8. 10.1038/nature13145. PMC4943525 24646994

[38] Regan JN, Lim J, Shi Y, Joeng KS, Arbeit JM, Shohet RV, et al. Up-regulation of glycolytic metabolism is required for HIF1α-driven bone formation. PNAS. 2014;111:8673–8. 10.1073/pnas.132429011. PMC4060724 24912186

[39] Deng LM, Feng ZH, Zheng HY, Li XJ, Wu X, Quan WY, et al. Isolation and characterization of a novel homopolysaccharide (SFP-1) from *Sargassum fusiforme*: Promising anti-osteoporosis activity by modulating adipo-osteogenic differentiation. Ind Crops Prod. 2024;207:117749. 10.1016/j.indcrop.2023.117749.

[40] Wang SQ, Li G, Zhang XF, Wang YQ, Qiang Y, Wang BL, et al. Structural characterization and antioxidant activity of *Polygonatum sibiricum* polysaccharides. Carbohydr Polym. 2022;291:119524. 10.1016/j.carbpol.2022.119524. 35698327

[41] Zhou LS, Jiao YK, Tang JY, Zhao ZZ, Zhu HY, Lu Y, et al. Ultrafiltration isolation, structure and effects on H1N1-induced acute lung injury of a heteropolysaccharide from *Houttuynia cordata*. Int J Biol Macromol. 2022;222:2414–25. 10.1016/j.ijbiomac.2022.10.027. 36252625

