# Supplementary material for: Isolation, structural characterization of oligosaccharide AMO-1 from Avicennia marina fruit and its anti-osteoporotic effects via PI3K/AKT/HIF‑1α signaling pathway activation
Source: Nat Prod Bioprospect. 2026 Oct 22;16(1):95. doi: 10.1007/s13659-026-00646-8 (PMC13691021; doi:10.1007/s13659-026-00646-8)
Supplement: Supplementary file 1 [file 13659_2026_646_MOESM1_ESM.docx]

Isolation, structural characterization of oligosaccharide AMO-1 from *Avicennia marina* fruit and its anti-osteoporotic effects via PI3K/AKT/HIF‑1α signaling pathway activation

Lishuang Zhou ^a,1^, Zhenhui Feng ^a,1^, Yijing Zhang ^a^, Lei Tan ^a^, Pan Zhou ^a^, Xia Wu ^a^, Xing Chen ^d^, Qinyuan Zhang ^a^, Qiwen Cai ^a^, Liutong Yu ^a^, Kefeng Wu ^a^^,b,c,*^, Luming Deng ^a,b,c,*^

^a^ *The Marine Biomedical Research Institute of Guangdong Zhanjiang, School of Ocean and Tropical Medicine, Guangdong Medical University, Zhanjiang, 524023, PR China*

^b^ *Guangdong Engineering Technology Research Center for the Development and Utilization of Mangrove Wetland Medicinal Resources, Guangdong Medical University, Zhanjiang, 524023, PR China*

^c^ *College of Tropical Southern Medicinal Herbs and Marine Organism Utilization, Guangdong Medical University, Zhanjiang, 524023, PR China*

^d^ *Department of Environmental Science and Engineering, Fudan University, Shanghai, 200438, PR China*

^*^ Corresponding authors: winokhere@sina.com (K. Wu), lumingdeng@gdmu.edu.cn (L. Deng)

^1^ These authors contributed equally to this work.

***Mono******saccharide composition analysis***

The monosaccharide composition of AMO-1 was determined using ion chromatography. As shown in **Fig. 2D-F**, the results indicated that AMO-1 was composed of three monosaccharides: galactose (Gal), glucose (Glc), and fructose (Fru). Given that Fru was prone to degradation under prolonged high-temperature conditions, and the 2-position carbonyl group of Fru can be reduced during hydrolysis to yield glucose and mannitol, making the quantification of fructose challenging. Therefore, we employed two different hydrolysis conditions to calculate the molar ratios of the monosaccharides. According to the standard curve calculations, in the 120 ℃ hydrolysis method, the detected masses of Gal and Glc in AMO-1 were 341.57 μg/mg and 218.15 μg/mg, respectively (**Fig. 2E**). In the 60℃ hydrolysis method, the detected mass of Fru in AMO-1 was 208.03 μg/mg (**Fig. 2F**). Integrating the results from both hydrolysis conditions, the molar ratios of Gal, Glc, and Fru were calculated as follows:

The ratio of Gal = 341.57/ (341.57+218.15+208.03) * 100%/ 180.16 = 0.25;

The ratio of Gal = 218.15/ (341.57+218.15+208.03) * 100%/ 180.16 = 0.16;

The ratio of Fru = 208.03/ (341.57+218.15+208.03) * 100%/ 180.16 = 0.15.

Therefore, the actual monosaccharide ratio of AMO-1 is Gal: Glc: Fru = 0.25 :0.16: 0.15.

**Fig. S1**. UV spectrum of AMO-1.

**Fig. S2**. FT-IR spectrum of AMO-1.

**Fig. S3**. DEPT-135 NMR spectrum of AMO-1.

**Fig. S4**. SEM images of AMO-1 ((A): 500 ×, (B): 2000 ×) at different magnifications.

**Fig. S5.** Effect of AMO-1 on cell viability.

**Fig. S6.** H&E staining analysis of the (A) liver, (B) spleen, and (C) kidney of experimental mice (200×, scale bar: 100 µm). The red arrow shows a small amount of inflammatory cell infiltration in the spleen, and the green and blue arrows show glomerular atrophy and tubulo-tubular vacuolar degeneration in the kidney.

Original blot image for COL1 protein in Fig. 7 of the manuscript.

Original blot image for RUNX2 protein in Fig. 7 of the manuscript.

Original blot image for OCN protein in Fig. 7 of the manuscript.

Original blot image for OSX protein in Fig. 7 of the manuscript.

Original blot image for L1CAM protein in Fig. 9 of the manuscript.

Original blot image for TLE1 protein in Fig. 9 of the manuscript.

Original blot image for DDX5 protein in Fig. 9 of the manuscript.

Original blot image for P-PI3K protein in Fig. 9 of the manuscript.

Original blot image for P-AKT protein in Fig. 9 of the manuscript.

Original blot image for PI3K and AKT proteins in Fig. 9 of the manuscript.

Original blot image for HIF-1α proteins in Fig. 9 of the manuscript.

**Table S1.** The effect of AMO-1 on bodyweight and organ indexes in OP mice.

|  | **Days** | **CTRL** | **AMO-1**  **200 mg/kg** | **Dex (30 mg/kg)** | | |
| --- | --- | --- | --- | --- | --- | --- |
|  |  |  |  | **--** | **Cal**  **60 ng/kg** | **AMO-1**  **200 mg/kg** |
| **Body**  **weight (g)** | 0 day | 24.93±0.33 | 24.83±0.41 | 24.95±0.35 | 24.95±0.31 | 24.82±0.35 |
|  | 7^th^ day | 25.13±0.33 | 24.88±0.33 | 24.60±0.32 | 24.68±0.26 | 24.51±0.31 |
|  | 14^th^ day | 25.43±0.30 | 25.26±0.40 | 24.33±0.50 | 23.97±0.34 | 24.69±0.73 |
|  | 21^st^ day | 25.90±0.33 | 24.19±0.66 | 24.10±0.32**^#^** | 23.29±0.34 | 23.86±0.58 |
|  | 28^th^ day | 26.07±0.28 | 24.66±0.41 | 24.03±0.29**^#^** | 23.33±0.35 | 23.97±0.63 |
|  | 35^th^ day | 24.79±0.31 | 23.73±0.55 | 23.30±0.45 | 22.22±0.40 | 22.79±0.69 |
|  | 42^nd^ day | 25.19±0.26 | 23.50±1.02 | 23.24±0.37 | 22.60±0.40 | 23.29±0.94 |
|  | 49^th^ day | 25.40±0.39 | 22.77±0.64 | 22.99±0.41**^#^** | 22.07±0.38 | 22.70±0.76 |
|  | 56^th^ day | 25.08±0.33 | 23.93±0.95 | 22.91±0.39**^#^** | 22.09±0.46 | 22.41±0.72 |
|  | 63^rd^ day | 24.53±0.38 | 23.92±0.73 | 21.89±0.49**^##^** | 20.93±0.42 | 21.44±0.71 |
| **Organ**  **Index (%)** | Liver | 3.47±0.11 | 4.16±0.19 | 4.68±0.09**^####^** | 4.28±0.07 | 4.49±0.15 |
|  | Spleen | 0.28±0.01 | 0.32±0.04 | 0.22±0.03 | 0.20±0.01 | 0.23±0.01 |
|  | Kidney | 1.32±0.06 | 1.21±0.04 | 1.21±0.03 | 1.22±0.04 | 1.23±0.01 |

The data were analyzed using a one-way ANOVA and expressed as mean ± S.E.M. (n = 8/group) **^#^***p*< 0.05, **^##^***p* < 0.01, **^###^***p* < 0.001 and **^####^***p* < 0.0001 *vs.* CTRL mice.

**Table S2.** The differential proteins of femur among experimental mice.

| **Protein molecule** | **Group1** | | | | **Group2** | | | | **Group3** | | | |
| --- | --- | --- | --- | --- | --- | --- | --- | --- | --- | --- | --- | --- |
|  | **1** | **2** | **3** | **4** | **1** | **2** | **3** | **4** | **1** | **2** | **3** | **4** |
| Ighv1-59 | 624.273 | 355.363 | 225.105 | 669.926 | 180.500 | 204.281 | 136.346 | 89.457 | 246.028 | 278.844 | 480.900 | 335.258 |
| Ddx5 | 504.434 | 435.886 | 772.245 | 305.170 | 166.430 | 250.623 | 215.947 | 145.800 | 309.516 | 351.997 | 309.516 | 267.035 |
| Tmem192 | 193.579 | 153.068 | 192.579 | 234.091 | 94.855 | 94.049 | 93.243 | 93.049 | 149.228 | 173.140 | 180.764 | 219.924 |
| Osbpl7 | 239.171 | 187.887 | 290.454 | 238.171 | 126.121 | 102.088 | 85.545 | 104.585 | 149.989 | 165.071 | 175.855 | 169.369 |
| Tle1 | 124.502 | 138.078 | 125.502 | 110.925 | 61.019 | 68.074 | 87.146 | 79.802 | 155.301 | 239.610 | 194.693 | 179.608 |
| Atp9b | 325.710 | 326.710 | 285.153 | 366.267 | 122.383 | 192.240 | 284.170 | 170.168 | 306.866 | 343.733 | 323.818 | 400.515 |
| Drc7 | 889.784 | 697.346 | 1227.366 | 744.640 | 518.108 | 409.974 | 502.519 | 405.047 | 775.259 | 640.959 | 547.638 | 599.979 |
| Mios | 107.982 | 161.434 | 213.567 | 163.141 | 49.426 | 89.160 | 105.686 | 88.778 | 140.072 | 191.057 | 111.285 | 141.372 |
| Med22 | 81.004 | 63.037 | 80.004 | 98.970 | 60.489 | 16.197 | 51.031 | 42.572 | 158.685 | 171.815 | 158.685 | 145.556 |
| Tmub1 | 546.954 | 492.040 | 687.477 | 697.029 | 372.170 | 339.485 | 364.047 | 282.237 | 540.528 | 648.418 | 488.649 | 447.583 |
| Pex7 | 287.209 | 219.770 | 235.463 | 199.408 | 188.234 | 114.286 | 184.182 | 85.445 | 299.413 | 184.122 | 332.797 | 292.134 |
| Kbtbd2 | 78.602 | 64.453 | 77.602 | 92.750 | 34.138 | 55.853 | 77.568 | 56.853 | 154.319 | 107.807 | 83.662 | 85.440 |
| Cmc2 | 356.575 | 503.665 | 346.894 | 445.110 | 215.095 | 210.837 | 290.188 | 221.288 | 342.344 | 299.358 | 457.641 | 538.738 |
| C1galt1c1 | 207.034 | 141.583 | 217.899 | 171.872 | 103.920 | 108.660 | 111.247 | 95.308 | 180.202 | 131.131 | 162.871 | 177.281 |
| L1cam | 206.961 | 199.095 | 173.302 | 150.745 | 99.351 | 70.100 | 98.093 | 129.859 | 152.599 | 150.929 | 232.121 | 234.724 |
| Mras | 393.369 | 357.943 | 358.943 | 322.518 | 219.797 | 164.913 | 274.681 | 229.797 | 319.710 | 279.713 | 359.707 | 318.710 |
| Pcbp2 | 435.665 | 257.791 | 334.869 | 311.152 | 262.818 | 256.772 | 210.276 | 225.893 | 313.048 | 257.521 | 316.858 | 307.645 |
| Ppt1 | 1089.224 | 1215.944 | 1202.413 | 1400.310 | 806.185 | 700.896 | 1080.660 | 1024.748 | 1317.637 | 1311.010 | 1161.403 | 1052.219 |
| Ralgapb | 302.794 | 287.710 | 352.561 | 294.080 | 254.178 | 219.393 | 291.513 | 242.617 | 308.050 | 325.548 | 274.294 | 318.405 |

Group 1: CTRL mice; Group 2: vehicle-treated OP mice; Group 3: AMO-1-treated OP mice.
